# Golden Ratio and Cybernetic Aesthetics for Hidden Patterns in Repetitive Human Movements: Golden-faux-sinus as a Two-Dimensional Optimization Model

**DOI:** 10.1007/s00422-026-01050-8

**Published:** 2026-07-31

**Authors:** Cristiano Maria Verrelli, Lucio Caprioli, Cristian Romagnoli, Mohamed El Arayshi, Maciej Ryszczuk, Marco Iosa

**Affiliations:** 1 Electronic Engineering Department, University of Rome Tor Vergata, Via del Politecnico 1, Rome, 00133 Italy; 2 Sports Engineering Laboratory, Department of Industrial Engineering, University of Rome Tor Vergata, Via del Politecnico 1, Rome, 00133 Italy; 3 Human Performance, Sport Training, Health Education Laboratory, Department of Human Science and Promotion of Quality of Life, San Raffaele Open University, Via di Val Cannuta, Rome, 00166 Italy; 4 S & C coach, Physiotherapist, Warsaw, Poland; 5 Department of Psychology, Sapienza University of Rome, Rome, Italy; 6 Smart Lab, IRCCS Santa Lucia Foundation, Rome, Italy

**Keywords:** Two-Dimensional Optimization, Fluidity, Technique Learning, Praxis System, Generalized Fibonacci sequence, Mechanization, Self-similarity, Golden Ratio, Tennis forehand, Jerk minimization

## Abstract

**Abstract:**

It is not easy to measure the amount of information within an object that is capable of leading to aesthetics-based feelings in a viewer, especially because of a subjective variability of human perception. Cybernetic aesthetics, for its part, might provide a rational way for understanding such a human phenomenon at least in special cases. Now, cyclic human movements — not only gait cycles, but also swimming strokes and tennis forehand strokes — were found, with their given internal sub-phases, to be characterized, from a temporal point of view, by the existence of coordinatively mechanized and self-similar harmonic fractal patterns. Such harmonic patterns were specifically found to be symmetrized in time so as to compose a generalized Fibonacci sequence and then implicitly dictated by the golden ratio, when it occurs as the ratio of the resulting sub-phases durations. The lowest amount of information for the movement temporal design (Shannon entropy minimization, SEM) makes one sub-phase duration of the movement temporally generate an entire sequence of sub-phase durations of the same movement. However, different spatial profiles can correspond to identical temporal partitions, suggesting the presence of an additional optimization principle that acts within the spatial domain. The original contribution of this paper consists of unveiling what is beyond the aforementioned temporal characterization, revealing the existence of a possible cyclic movement attractor, named golden faux sinus, which is based on a spatial smooth sewing of zero-jerk portions. This might suggest how the neuromuscular system organizes complex repetitive movements and how information processing is exploited into a sub-movement generation architecture. The resulting hidden patterns are the ones that might be caught by a viewer, who is able to concentrate separately on different categories of meaning. Experiments concerning young healthy walking subjects, as well as ATP-WTA top-level tennis players during some of the latest strongest moments of their career, illustrate the effectiveness of the presented derivations.

**Graphic Abstract:**

**Figure:**
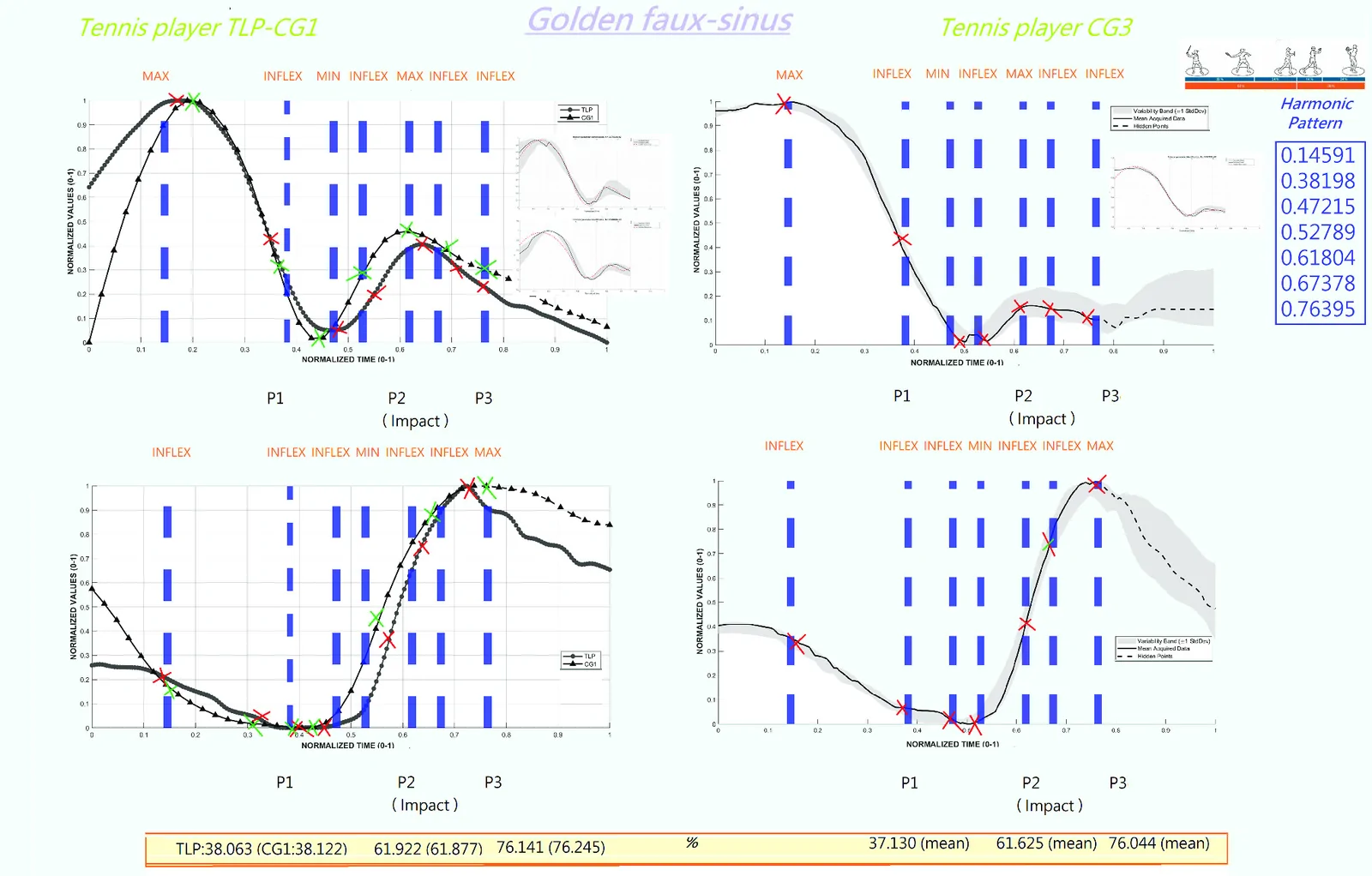

## Introduction

Cybernetics is originally defined as the study of self-regulating systems (biological, social, or technological) that achieve or maintain specific goals through the use of information, by allowing for effective and adaptive self-correction. More in particular, cybernetic aesthetics (Franke 1977) is based on the premise that the perception of complex structures offers satisfaction. One of those complex hidden structures might involve the features of a repetitive movement (especially when it is connected to the golden ratio $\phi =(1+\sqrt{5})/2 \approxeq 1.618$, namely, the ratio between the diagonal and the side of a regular pentagon or the positive solution to $x^{2}=1+x$ within the problem of dividing a line segment such the ratio of the whole segment to the longest part is equal to the ratio of the longest part to the shortest). The way they stimulate emotional reactions in viewers is a challenging topic. In the cybernetic sense, emotions are consciousness-awakening signals that direct attention to specific situations of importance. The interest of viewers is certainly stimulated by the search for hidden information, like in a puzzle (Franke 1977), which can evoke feelings by association. Even though the human brain is able to receive information through the senses at a high rate, only a small portion of bits per second can be received consciously. During transmission to the brain neuronal network, the information received by the sense receptors is processed with the aim of removing what is unimportant, while selecting and decoding what is important. In light of cybernetics, consciousness is thus the temporary storage of information. However, a human being is able to concentrate separately on several categories of meaning. In viewing pictures, one might give attention to the geometrical aspects or to the colour arrangement, as well as one may consider the historical, metaphorical and allegorical meaning of shapes (Franke 1977). A picture or vision, in which the amount of information in each category does not exceed a relatively small number of bits while consisting of several categories, will maintain interest in it, especially when relationships between the information contained in the various categories of meaning exist. In this way, a picture can have a relatively large number of bits of information without overtaxing viewers. As aforementioned, the golden ratio is the irrational number ϕ, likely first identified by the Ancient Greeks. It emerged in both geometrical contexts and in human anthropometry, where it approximates the ratio between a person’s height and the distance from the navel to the feet (see the subsequent Section 5). The Greeks had quickly recognized this proportion as a hallmark of harmonic structures and incorporated it into their artistic and architectural endeavours, including sculptures such as the Venus of Milos and buildings like the Parthenon. In the later centuries, its presence was identified in various scientific fields such as botany and psychology. It was also further embraced in the arts by (Iosa et al. 2018). Further mathematical properties of the golden ratio were uncovered (notably by Kepler), and, in particular, its connection with the Fibonacci sequence. Generalized Fibonacci sequences (Horadam 1961) (see also (Bormashenko 2022)) are sequences of numbers that are generated by the first two elements (called seeds) *via* the following generation rule: from the third number, every element of the sequence is the sum of the previous two (Verrelli et al. 2021a, b). Starting from their use in symmetric walking – where the stance duration (third element of the sequence) is the sum of the double support and swing durations (first and second elements of the sequence, respectively) and, in turn, the duration of the entire gait cycle (fourth element of the sequence) is the sum of the stance and swing durations –, generalized Fibonacci sequences were discovered to extend the resulting original walking gait characterization to completely automatized gestures in elite swimmers and tennis-players (Verrelli et al. 2021a, b). In particular, a longer Fibonacci sequence is associated with a higher level of coordinative mechanization in the generation of the event, since the inclusion of a simpler subphase in the sequence removes an aggregate phase from the set of seeds and makes an elementary physical phase enter it. In this light, coordinative mechanization is viewed as an increase in the number of physical phases of the movement that enter the set of seeds within the self-referential loop so as to reduce the number of durations to be independently determined and memorized in the design of the event. This happens at the price of an increased number of repetitions performed by the human, in which the temporal symmetrization step is successfully performed. Now, it is true that swimming does not seem to belong to Central Pattern Generator-based instinctive patterns, but it naturally owns a rhythmicity similar to walking and running as soon as it is induced by repetitive training for a long enough time; on the other hand, several aspects of tennis movements certainly become highly stereotyped and efficient with practice, with the forehand stroke turning out to start and end at similar postural configurations while being repeated cyclically in a rally. In alignment with the aforementioned discussion, the golden ratio was found to characterize hidden self-similar patterns: all the ratios between two consecutive elements of the sequence (namely, the durations of the sub-phases of the above movements) are surprisingly equal. The temporal partition of a movement into phases that maintain the same proportion in their ratios appears to be an optimization strategy, namely, SEM (Shannon entropy minimization) (Verrelli et al. 2025), adopted to address the motor equivalence problem associated with the coordinated control of numerous degrees of freedom. Indeed the previously described mechanisms account for the harmonic temporal organization of movement. They are not able to explain how spatial trajectories – such as limb paths – respecting the self-similar timing are shaped to produce fluid and non-redundant patterns. Since, different spatial profiles might give rise to identical temporal partitions, a second optimization rule should underlie the spatial dimension of human movement. As far as several categories of meaning in a picture are concerned, along with the relationships between the information contained in the various categories of meaning, here, a spatial category has to exist beyond the temporal one while being meaningfully related to it. The question thus is: what spatial optimization principle, beyond the temporal Shannon entropy minimization, might govern the fluid and non-redundant trajectories of cyclic human movements, and how does this principle might relate to the golden ratio-based temporal partition? Such a question is somehow associated with the praxis system (Hurt 2016) that concerns cognitive and motor processes enabling skilled movements interacting with the environment. It is also associated with the approach of (Flash and Hogans 1985) and (Assila et al. 2026), in which the question regarding what basis the central nervous system selects one specific trajectory from the infinite number of possible ones is posed. As noticed there, investigations of multi-joint movements may provide considerable insight into the strategies employed by the central nervous system in the control of skilled activities.

The original contribution of this paper^1^, whose most technical description is separately reported in Appendices A and B for the sake of readability, is to provide an answer to the previously posed open question. Using human gait as an archetypal cyclic and harmonic movement, we propose a cyclic movement attractor that governs spatial coordination, at a timing complying with SEM (T-SEM), through the generation of maxima-minima in the space domain and the T-SEM-induced sub-phase jerk minimization & smooth transition among the T-SEM-induced sub-phases, with jerk impulsive actions occurring at the T-SEM-induced sub-phase transition instants (see (Pietrosanti et al. 2025) for torque-producing impulsive EMG activities for upper limb muscles during walking gaits). Such a second optimization dimension, besides the time-optimization one (suitably enlarged here to capture a more complete movement understanding), is assumed to be related to the movement space-shaping and fluidity enhancement. In conjunction with the first optimization procedure, it leads to the here defined *golden faux-sinus*, namely, a special function that harmonically resembles a sinus function multiplied by an exponential. Passing, by transposition, through the front crawl stroke of a top-level swimmer, the same mechanism is then meaningfully discovered within the tennis forehand strokes of ATP-WTA top-level tennis players during some of the latest strongest moments of their career, demonstrating its generality across different repetitive motor domains. It is hypothesized that the described procedure might identify a unified two-dimensional framework through which the human motor system achieves elegant, efficient, and highly technical automatized gestures. It is true that alternative cost functions could be proposed, including minimum torque change, minimum variance, and metabolic energy minimization. However, the piecewise constant-acceleration solution of this paper is a specific instance of a minimum-jerk trajectory with jerk impulses at transitions to maximize predictability of trajectories and produce smooth, graceful movements (see (Flash and Hogans 1985) and (Assila et al. 2026)). It provides a null hypothesis for the spatial shape of harmonic movements, which empirical data can be compared to. It is worth noting that the movements analyzed here – particularly the tennis forehand – are short in duration and highly coordinative, involving sequential activation of lower limbs, trunk, upper limb, and racquet. In such explosive, brief gestures, the overall metabolic energy consumption of a single stroke is negligible compared to the cognitive and neuromuscular demand of synchronizing multiple degrees of freedom. Hence, coordinative smoothness (as reflected by jerk minimization) likely prevails over pure energy minimization as a performance criterion. This view aligns with studies on motor expertise showing that skilled performers prioritize movement timing, coordination, and predictability over energetic efficiency in short-duration, high-accuracy tasks (Lohse et al. 2014; Sparrow and Newell 1998). The present model thus focuses on jerk as a proxy for movement fluidity and coordinative quality, rather than as a direct measure of metabolic cost.

## Materials and Methods

**Fig. 1:**
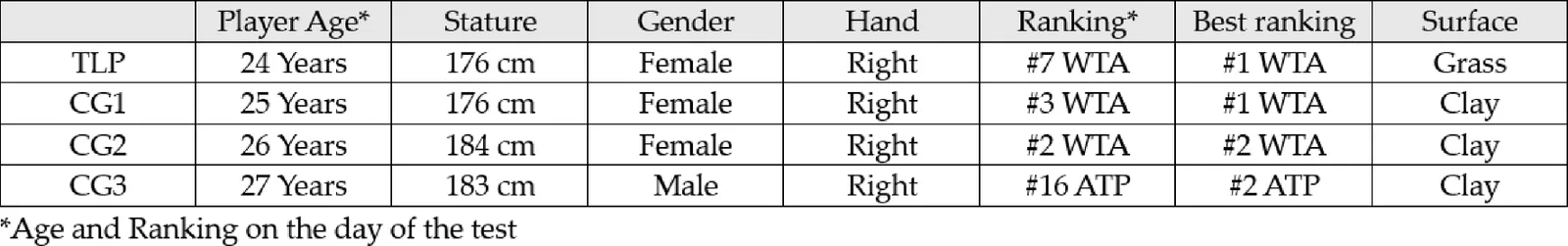
General data for the top-level tennis players TPL, CG1, CG2, CG3.

**Fig. 2:**
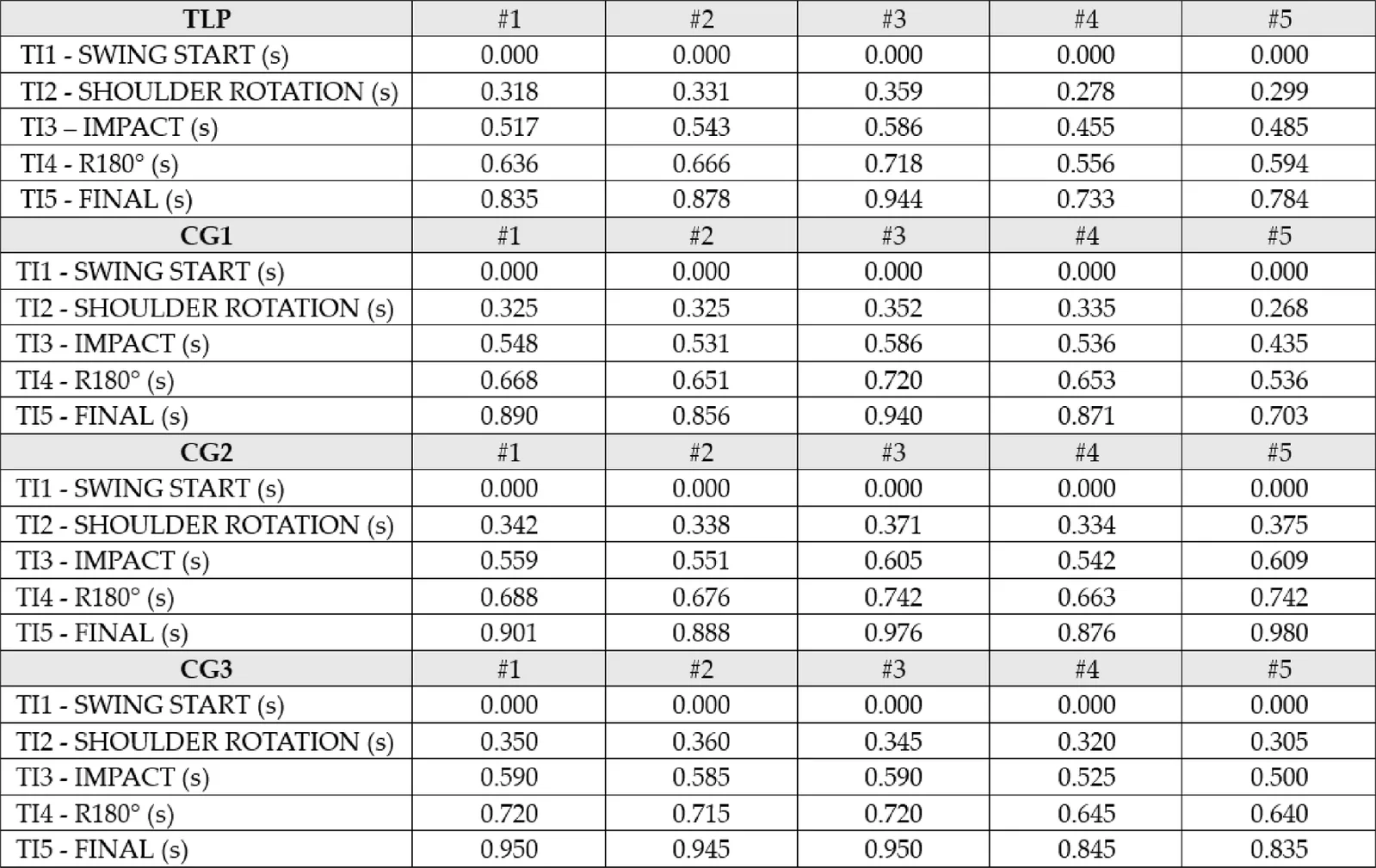
Time instants TI1-TI5 for the top-level tennis players TPL, CG1, CG2, CG3.

The concepts developed within this paper will be illustrated by experimental results concerning forehand strokes of top-level tennis players during some of the latest strongest moments of their career, passing through gait cycles of young healthy subjects walking at a comfortable speed^2^ in Section 3.1 and a front crawl stroke of a top-level swimmer^3^ in Section 3.2. A top-level tennis player TLP [Female, 24 yrs, 176 cm, #7 WTA (best ranking #1 WTA)] is first engaged. The investigation is conducted on video recordings (see Appendix C for an inter-reliability analysis) acquired by the coach of the top player during common tennis practice sessions of an international tennis tournament. The study involves forehand shots played in a practice rally situation, recorded from the lateral perspective by a camera placed on a tripod at a height of 1.20 m above the ground. A control group of rally shots carried out, on the clay court, by three ATP-WTA top-level tennis players CG1, CG2, CG3 (CG1 is the same athlete TLP during a different international tennis tournament) is also relevantly considered. General data for TPL and CG1, CG2, CG3 are summarized in Figure 1. The videos collected for analysis are captured in quality HD, 240 fps. The sub-phases of each forehand stroke of the player are identified, within the videos, in accordance with the definition of the time-instants TI1: swing start point of maximum loading (point of maximum racquet height); TI2: shoulder rotation, starting instant of the shoulder line rotation; TI3: impact; TI4: R180, 180 deg rotation of the racquet after impact (the point where the racquet cap faces the camera); TI5: final, time instant when the kinetic energy of the blow is exhausted. Such five time instants (TI1–TI5) had been initially defined on the basis of established biomechanical descriptions of the tennis forehand. Their operationalization in the video analysis had been reviewed and approved by two certified tennis coaches. This dual verification ensured that the identified events correspond to functionally meaningful and reproducible phases of the stroke. Figure 2 reports the time instants TI1-TI5 corresponding to five (rally) forehand strokes for TLP, CG1, CG2, CG3. Both the horizontal and vertical displacements of the hands of the subject within the image plane are also tracked from the instant TI1 to the end of the stroke (TI5). As far the horizontal profile is concerned, the axis is oriented to the left in the image plane (from the net to the backward of the court). As far the vertical profile is concerned, the axis is oriented upward. A marker is placed by the operator in correspondence with the player’s dominant hand, and an automatic tracking system is adopted within the open-source application Kinovea. In order to control the accuracy of the analysis and ensure proper tracking, the operator checks frame by frame the placement of the marker and, if needed, he/she manually corrects its position. The video analyses of all the shots are conducted by the same operator, and a sample of them is repeated by a second examiner to assess the reliability of the measurement. Once the tracking task is ended, the digitized coordinates are passed through a filtering procedure to remove noise. Two applications of a second-order Butterworth filter (with cutoff frequency 8 Hz, based on residual analysis) are carried out. To initialize the filter, the trajectory is extrapolated for ten data points on each side using reflected values around the end-points; extrapolated points are then removed from the filtered results (Smith 1989). Piecewise Cubic Hermite Interpolating Polynomial (PCHIP) is used to interpolate values missing due to occlusions (Rabbath and Corriveau 2019; Ghosh et al. 2025). Since horizontal and vertical hand positions, as next sections shall show, were normalized to the unit interval for each stroke individually (0 = minimum horizontal displacement, 1 = maximum), the absolute distance calibration was not required. To minimize parallax, only strokes in which the player’s body remained within a central lateral corridor (±1.5 m from the camera’s line of sight) were retained. Under this condition, changes in image scale due to depth variations were below 5%, as verified by tracking a stationary reference object at court corners.

## Temporal-Spatial Optimization

The main theoretical contribution of this paper is described in this section. The walking gait is first considered (subsection 3.1) for the sake of simplicity (with an interpretation in terms of the front crawl stroke of a top-level swimmer being provided in subsection 3.2) and then extended, by transposition, to the tennis forehand in subsection 3.3. Notice how the transposition of the temporal Fibonacci structure from walking to swimming and then tennis is based on the mathematical duality of the percentage durations derived from the self-similarity condition, not on a demonstrated biomechanical equivalence of the underlying neural or muscular synergies. This analogy is, in other terms, heuristic and generative.

### Starting from the walking gait

Consider the walking gait cycle of a healthy subject at a comfortable speed. Let ${\mathcal {F}}$ represent the gait cycle, ${\mathcal {F}A}$ represent the double support, ${\mathcal {F}B}$, ${\mathcal {F}C}$ represent the two swing phases. In light of the coordinative mechanization process and in accordance with Appendix D, the gait cycle is: 1. once-Fibonacci-extendable if the swing phases have the same duration; 2. twice-Fibonacci-extendable if the swing phase can be partitioned into two sub-phases, with the longest one lasting like the double support; 3. three-times-Fibonacci-extendable if the double support phase can be partitioned into two sub-phases, with the longest one lasting like the shortest sub-phase of the swing phase. Now, a straightforward computation shows that a three-times-Fibonacci-extendable gait cycle (take for simplicity duration 1), when self-similarly partitioned, so as to obtain the same ratio ϕ=1.61804 between two consecutive durations of the sequence and thus minimize the Shannon entropy, reads:

$$\begin{aligned} 0.09018, 0.14591, \overbrace{0.23608}^\mathrm{double \ support}, \overbrace{0.38198}^\textrm{swing}, \overbrace{0.61804}^\textrm{stance}, \overbrace{1}^\mathrm{gait \ cycle}. \end{aligned}$$

In particular: 1. the once-Fibonacci-extendability condition corresponds to a symmetrical gait cycle in which the percentage durations of both the swing phases are equal to 38.198%, the first starting at ${ 11.804}=23.608/2$% of the gait cycle, the second starting at ${ 61.806}=23.608+38.198$%; 2. the twice-Fibonacci-extendability condition corresponds to a partition of the swing phase (percentage-wise lasting 38.1984) with boundary event at 14.591% of such a swing phase (and thus at 76.395% of the gait cycle); 3. the three-times-Fibonacci-extendability condition corresponds to a partition of the double support phase with boundary event at 7.2955=14.591/2% of each of the two portions of the double support phase (the first occurring at 7.2955%, the second occurring at ${ 57.2975}=11.804+38.198+7.2955$%). Now, the partitions of the last two items do acquire physical significance as soon as they identify the knee flexion angle peak and the instant of the minimum position of the foot relative to the tibia with a 90 deg angle being plotted at 0 deg as boundary events, respectively. With this in mind, the following percentages, in order, are definitely recognized to be meaningful within the second half of the walking gait: 57.2975%, 61.804%, 76.395%, while the following percentages, in order, turn out to be meaningful within the first half of the walking gait: 7.2955%; 11.804%; 26.395%. In addition, the following points come from the three-times-Fibonacci-extendability condition applied to the sub-partition of each swing phase equalling the double support phase within the first and second swings: 40.982%; 90.982%. Taking the knee flexion angle profile, the key idea is then to: 1. associate sewing points with the percentages 57.2955%, 61.804%, and 90.982% of the knee flexion angle profile (second half of the walking gait), besides the maximum point already associated with the percentage 76.395%; 2. associate sewing points with the percentages 7.2955%, and 26.395% of the knee flexion angle profile (first half of the walking gait), besides the maximum and minimum points associated with the percentages 11.804% and 40.982%, respectively; 3. use elementary five time functions $a_{i}t^{2}+b_{i}t+c_{i}$, i=-1,0,1,2,3, for which jerk is minimized to zero (constant-acceleration sub-phase), to connect all the (consecutive) four sewing points and allowing for jerk impulsive actions at the sub-phase transition instants; 4. carry out a ${\mathcal {C}}^{1}$-sewing of the elementary time functions to obtain an overall continuous function, with continuous derivative, that reconstructs the knee flexion angle profile, provided that the final point equals the initial one for movement repetitivity.

**Fig. 3:**
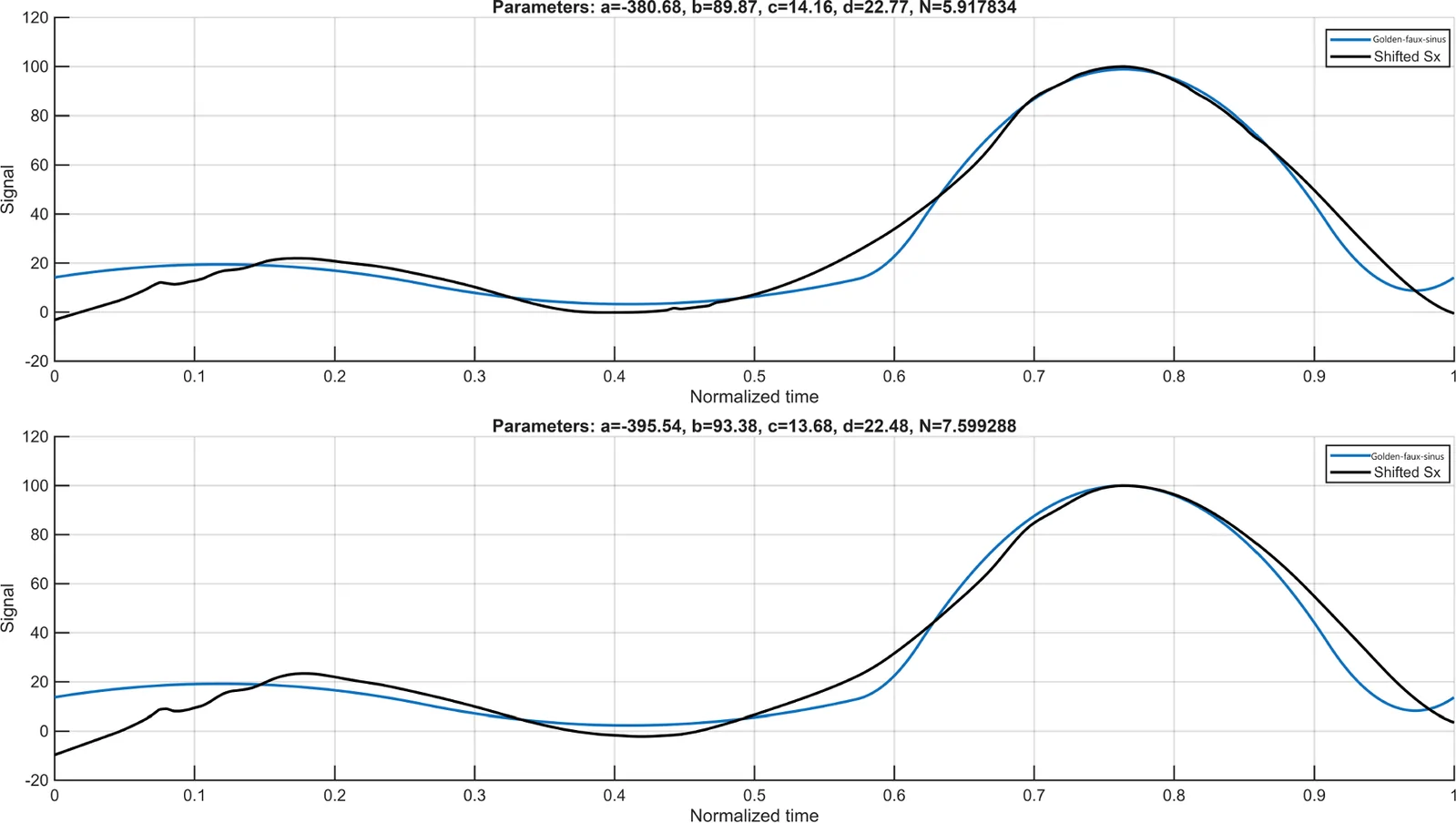
Healthy subject HS1: knee flexion angle profile in degrees [abscissa reporting the normalized time with respect to the total duration of the gait cycle] and *golden faux-sinus* for walking (reconstructed profile by (1)-(15) of Appendix A).

**Fig. 4:**
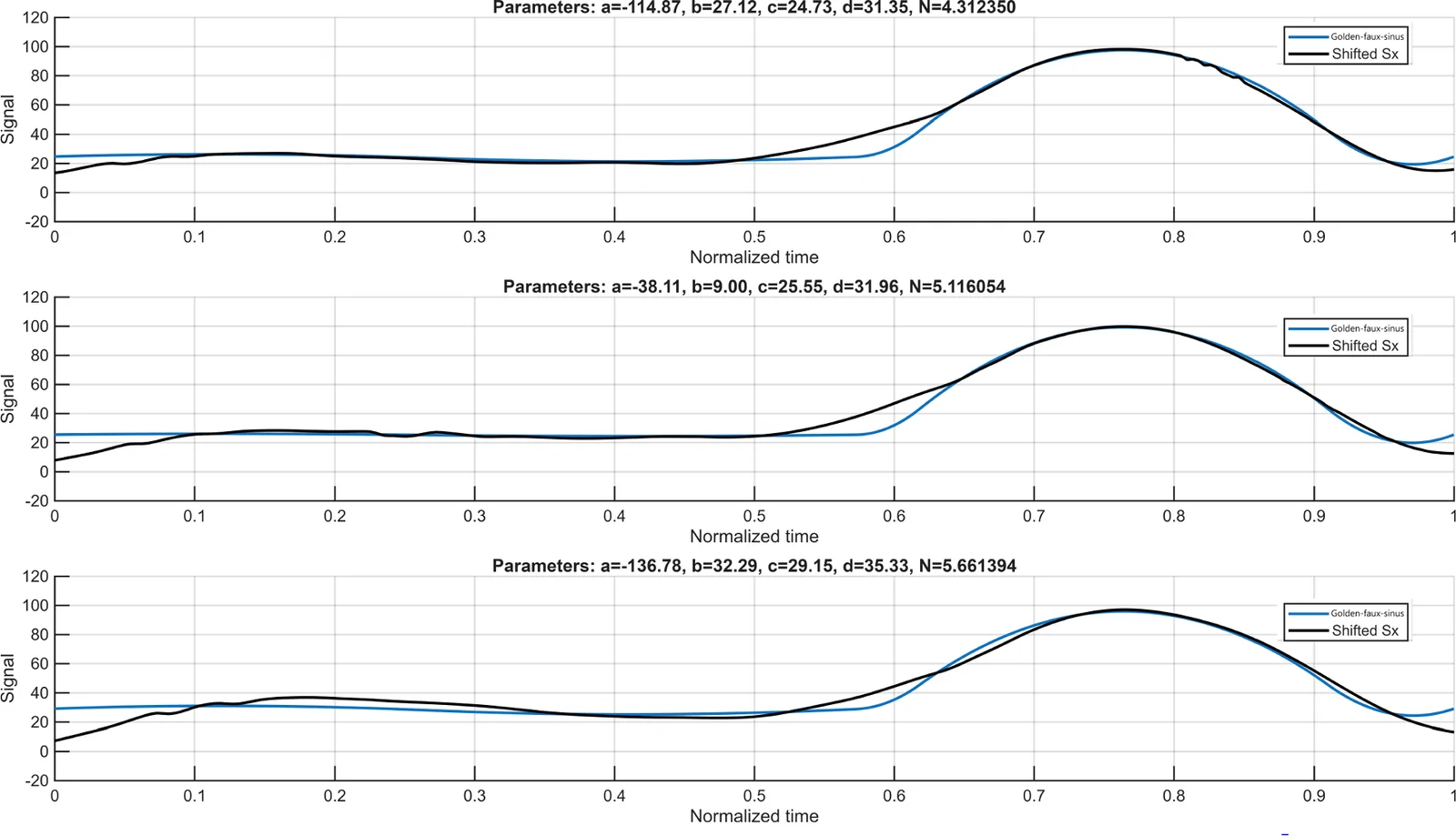
Healthy subject HS2: knee flexion angle profile in degrees [abscissa reporting the normalized time with respect to the total duration of the gait cycle] and *golden faux-sinus* for walking (reconstructed profile by (1)-(15) of Appendix A).

**Fig. 5:**
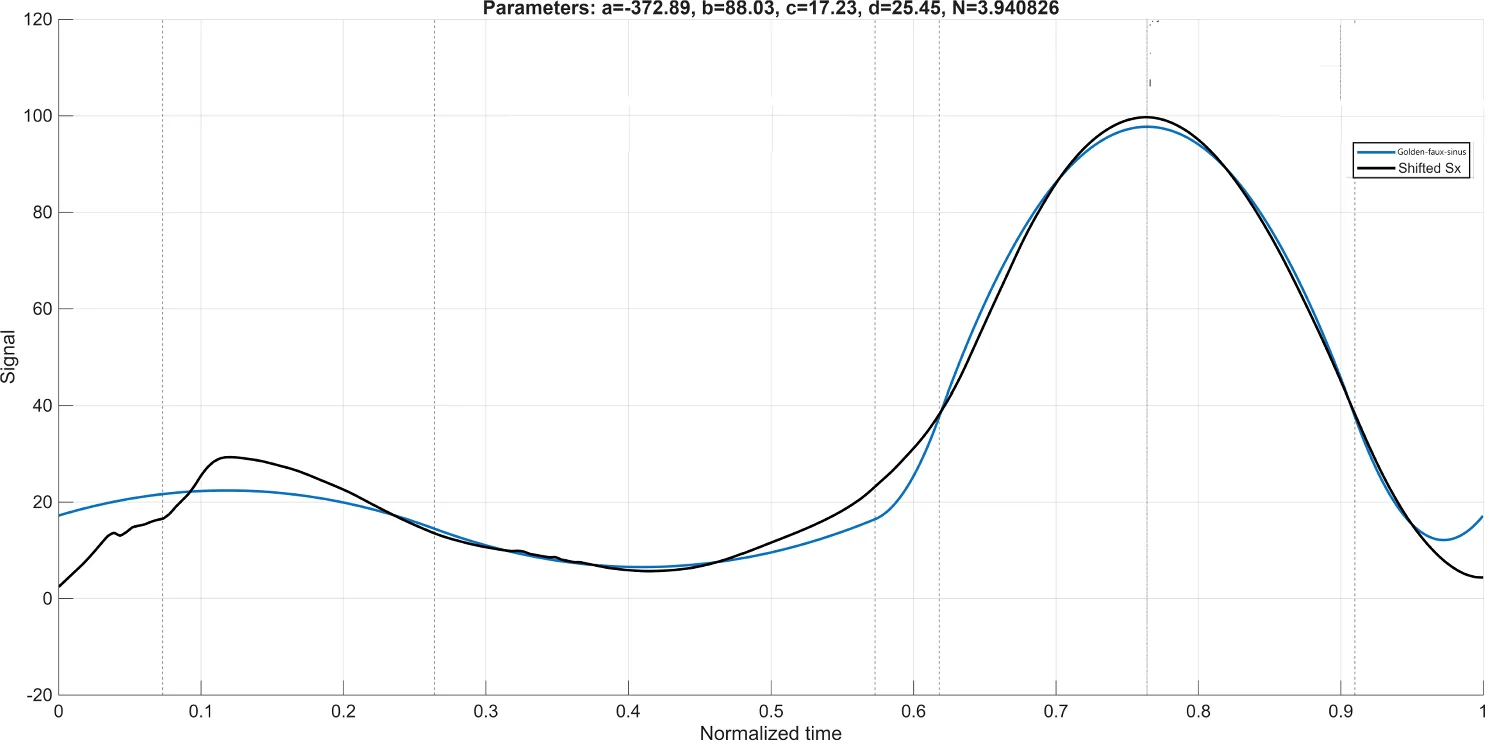
Healthy subject HS3: knee flexion angle profile in degrees [abscissa reporting the normalized time with respect to the total duration of the gait cycle] and *golden faux-sinus* for walking (reconstructed profile by (1)-(15) of Appendix A).

The resulting function, owing to the above alternation of maxima/minimum points and to the nature of the elementary basis functions, does have a mirrored shape that is similar to a biased sinus function multiplied by an exponential. Figures 3-5 report the *golden faux-sinus* for walking by (1)-(15) of Appendix A (see technical remarks in Appendix B and a technical description of the reconstruction algorithm in Appendix E) for three young healthy subjects HS1 (155 cm, 51 kg, 34 y.o., two gait cycles), HS2 (166 cm, 64 kg, 21 y.o., three gait cycles), HS3 (177 cm, 75.4 kg, 21 y.o., one gait cycle) exhibiting almost coordinatively mechanized and (symmetric) self-similar gaits in terms of double support, swing, stance: HS1: (25.261%, 37.375%, 62.625%), (25.026%, 37.487%, 62.512%); HS2: (25.202%, 37.399%, 62.601%), (23.077%, 38.462%, 61.538%), (22.359%, 38.820%, 61.180%); HS3: (22.008%, 38.996%, 61.004%). In order to better highlight the effectiveness of the second optimization principle, the knee flexion profiles have been subject to slight translations in order to make the global maxima coincide at the same instant. As Figures 3-5 show, the nature of the shape is fully captured in all the subjects, with horizontal translations ranging from 0.0034% to 0.0403% [HS1: 0.0403, 0.039; HS2: 0.0034, 0.0196, 0.028; HS3: 0.012]. The reconstruction errors *N* of Appendix E are relatively small [HS1: N=5.9178, N=7.599; HS2: N=4.3123, N=5.1160, 5.6613; HS3: N=3.9408] as explicitly reported in Figures 3-5. It is worth noticing how similar phase percentages lead to different reconstruction errors, illustrating how the second optimization principle acts differently from the first one.

### Passing thorugh the front-crawl stroke

**Fig. 6:**
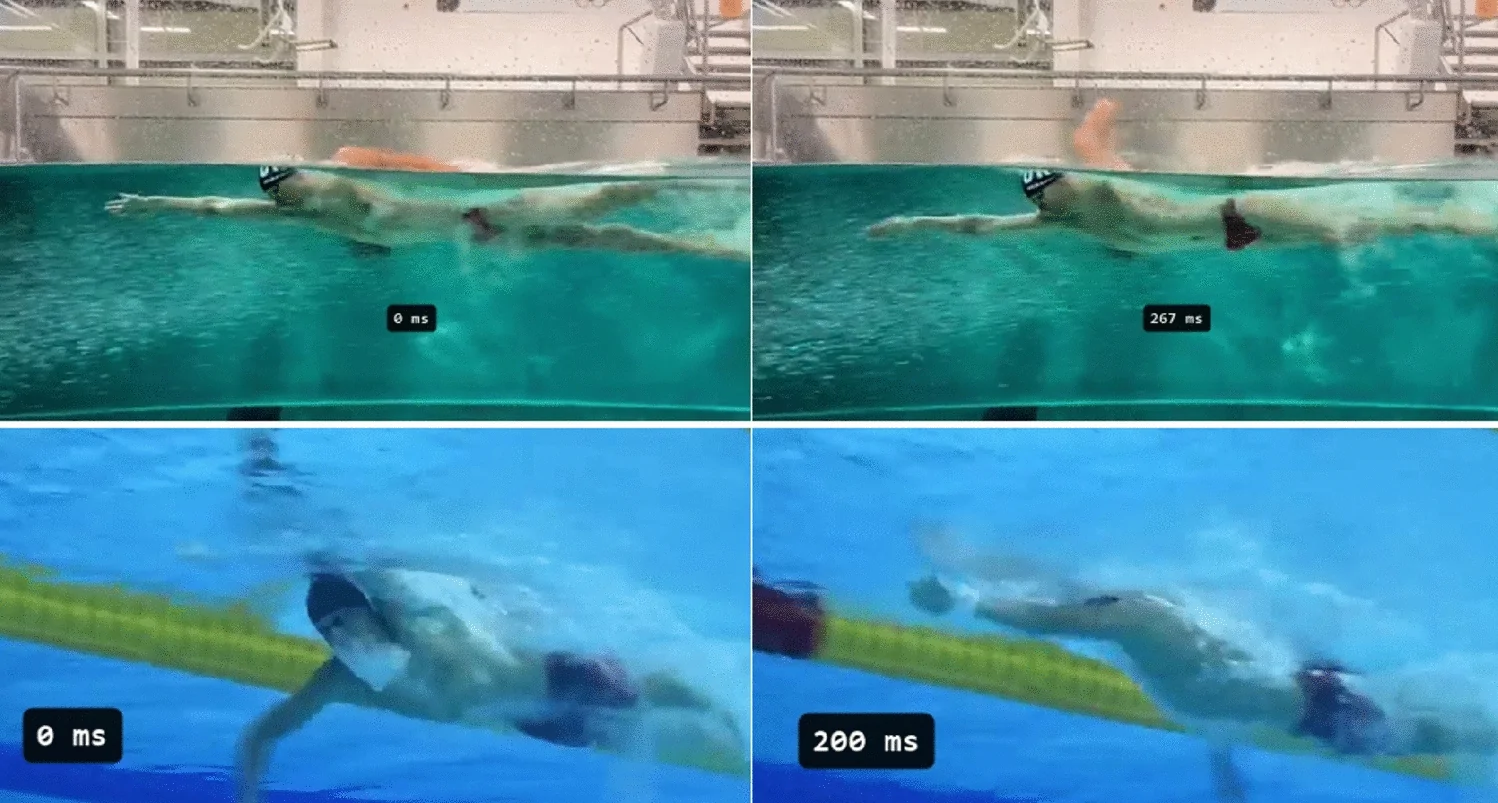
Top: spatial event (ascent to descent for the elbow) characterizing the bisection of the recovery phase. Down: spatial event (lateral movement of the hand) occurring 11.804% after the beginning of ES.

The spatial events, such as the knee flexion angle peak, whose occurrences were predicted, within the gait cycle, on the basis of the Fibonacci extendability conditions, suggest the adoption of the transposition of walking into swimming presented in (Verrelli et al. 2021b) to find out special events occurring within the front-crawl stroke of top-level swimmers. Now, according to Corollary 1 in (Verrelli et al. 2021b): RE is associated with the double support phase in walking and, analogously, its duration has to be bisected; IN & DC are associated with the swing phase in walking and, analogously, its duration has to be divided into two phases (with percentage durations 14.591% and 23.608%, respectively); UP & EX & ES are associated with the swing phase in walking and, analogously, its duration has to be divided into two phases (with percentage durations 23.608% and 14.591%, respectively). Such partitions by transposition suggest the presence of spatial events occurring at: the middle of the RE phase in order to bisect the RE phase; 11.804% after the beginning of the ES in order to get a 11.804% + 11.804% =23.608% and 14.591% partition. Analyzing – by the same analytical tools as in (Verrelli et al. 2021b) – the Youtube video of the front crawl stroke of a top-level (male) swimmer (1500 m-performance) [https:/www.thefrenchswimcoach.com/]– the reader is referred to Figure 6 – , two spatial events actually result: i) time instant (266.5 ms) identifying the passage from the ascent to the descent phases for the elbow in the RE phase (RE duration: 533 ms); ii) time instant identifying the lateral movement of the hand occurring 11.804% (200 ms) after the beginning of the ES (ES duration: 440 ms, percentage duration 26.19%).

### Landing on the tennis forehand stroke

**Fig. 7:**
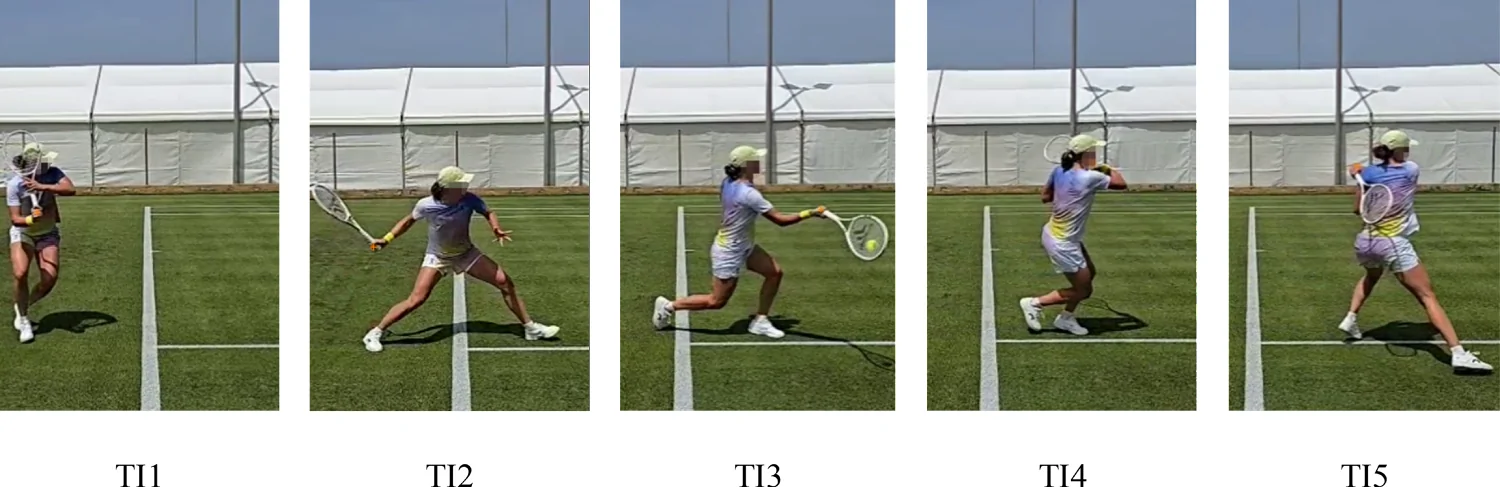
Time instants characterizing the forehand in tennis: swing start (TI1); shoulder rotation (TI2); impact (TI3); R180 (TI4); final (TI5).

We are able to finally and mainly extend the optimization principle to the tennis forehand stroke. As aforementioned, the forehand stroke is known to be characterized by five time instants: TI1, TI2, TI3, TI4, TI5, visualized for the sake of clarity in Figure 7. Such instants define four phases (the total duration PS of the stroke is the duration of the phase from TI1 to TI5): Phase *i*: from TI*i* to TIi+1, with duration P*i* (fraction of the forehand stroke duration PS), i=1,…,4.

In analogy to walking, if one starts from the 3-length generalized Fibonacci sequence: $\mathrm{P3 +P4}, \mathrm{P2+P1}, \textrm{1}$, then

- the 4-length generalized Fibonacci sequence P2, P3+P4, P2+P1, 1 is obtained under the constraint (once-Fibonacci-extendability): ${\mathcal {C}}_{1}$: P3+P4-P1=0;
- the 5-length generalized Fibonacci sequence P3, P2, P3+P4, P2+P1, 1 is obtained under the constraint (twice-Fibonacci-extendability): ${\mathcal {C}}_{2}$: P4-P2=0.

**Fig. 8:**
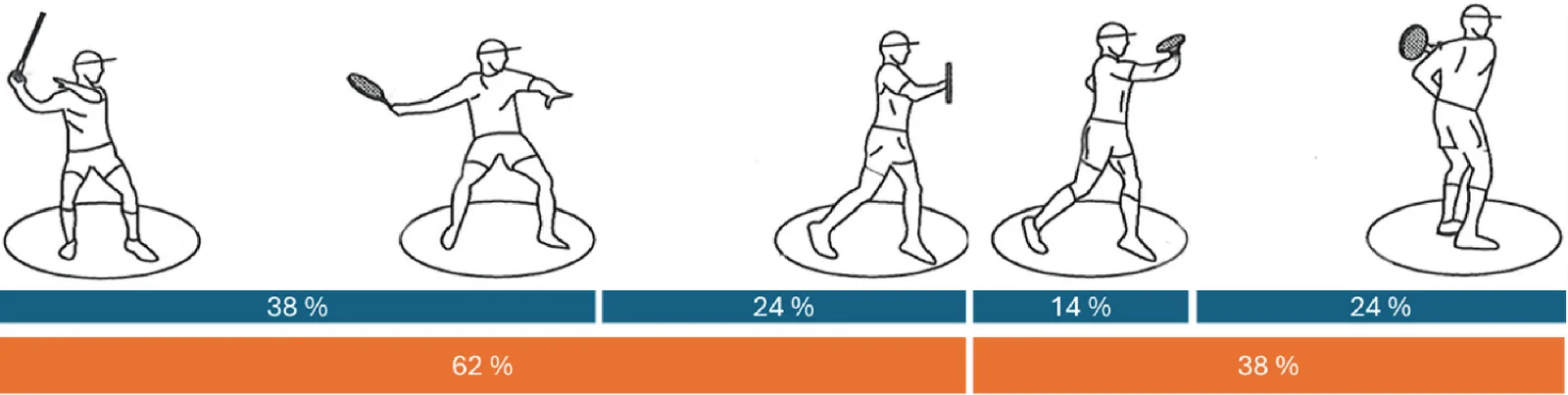
Harmonic partition (at a comfortable rally rhythm) of the stroke phases nullifying indices ${\mathcal {I}}_{f,4}$ and ${\mathcal {I}}_{f,5}$.

Indices simply quantifying harmonicity of tennis forehand strokes with reference to the 4-length and 5-length sequences above on the basis of TI1-TI5, are (see Figure 8)

$$\begin{aligned} {\mathcal {I}}_{f,4}= &  100\Big {[} \left( \mathrm{P1+P2} - 0.61804\right) ^{2} + \left( \textrm{P1} - 0.38198\right) ^{2} \\ &  \quad + \left( \textrm{P2} - 0.23608 \right) ^{2} \Big {]}^{1/2} \\ {\mathcal {I}}_{f,5}= &  100\Big {[} \left( \mathrm{P1+P2} - 0.61804\right) ^{2} + \left( \textrm{P1} - 0.38198\right) ^{2}\\ &  + \left( \textrm{P2} - 0.23608 \right) ^{2} + \\ &  \> \left( \textrm{P3} - 0.14591\right) ^{2} \Big {]}^{1/2}. \end{aligned}$$

The smaller such indices are, the stronger the level of self-similarity results.

Furthermore,

- the 6-length generalized Fibonacci sequence
  $$\begin{aligned} \textrm{P2S}, \ \ \ \textrm{P3}, \ \ \ \textrm{P2}, \ \ \ \mathrm{P3+P4}, \ \ \ \mathrm{P2+P1}, \ \ \ \textrm{1} \end{aligned}$$
  is obtained under the constraint (three times-Fibonacci-extendability) that the longest sub-phase of Phase 2 lasts like Phase 3 and the shortest one is denoted by 2S (with duration P2S).
- the 7-length generalized Fibonacci sequence
  $$\begin{aligned} {\mathcal {S}}: \ \textrm{P3S}, \ \ \ \textrm{P2S}, \ \ \ \textrm{P3}, \ \ \ \textrm{P2}, \ \ \ \mathrm{P3+P4}, \ \ \ \mathrm{P2+P1}, \ \ \ \textrm{1} \end{aligned}$$
  is obtained under the constraint (four times-Fibonacci-extendability) that the longest sub-phase of Phase 3 lasts like Phase 2S and the shortest one is denoted by 3S (with duration P3S).

Indeed, the self-similarity level of the four above sequences relies on the following original proposition, providing the harmonic percentages of the last Fibonacci sequence above.

#### Proposition 1

If P2S/P3S=ϕ, then the above sequence ${\mathcal {S}}$ exhibits an internal enhanced self-similar structure, with percentage duration of Phase 1 $\approxeq 38.198\%$, percentage duration of Phase 2 and Phase 4 $\approxeq 23.608\%$, percentage duration of Phase 3 $\approxeq 14.591\%$, percentage duration of Phase 2S $\approxeq 9.017\%$, percentage duration of Phase 3S $\approxeq 5.574\%$.

In summary, a four-times-Fibonacci-extendable forehand stroke (with duration 1), when self-similarly partitioned, so as to obtain the same ratio ϕ=1.61804 between two consecutive durations of the sequence and thus minimize the Shannon entropy, definitely reads:

$$\begin{aligned} &  \overbrace{0.05574}^\textrm{P3S}, \overbrace{0.09017}^\textrm{P2S}, \overbrace{0.14591}^\textrm{P3}, \overbrace{0.23608}^\textrm{P2}, \overbrace{0.38198}^\mathrm{P3+P4},\\ &  \overbrace{0.61804}^\mathrm{P2+P1}, \overbrace{1}^\mathrm{forehand \ stroke}. \end{aligned}$$

On the other hand, the partition leading to 2S and 3S does exhibit a physical meaning as soon as it comes from the minimum for the horizontal profile of the left-hand (non-dominant one) and the maximum for the vertical profile of the racquet-hand (dominant one), on the lateral camera plane (see Figs. 11,12,13,14,15). The correspondence between the profile of the knee in walking and the hand in tennis is assumed here, given the presence of the racquet as an extension of the limb in tennis and the contributions in literature that associate, from an evolutionary point of view, the characteristics of similar joints in quadrupedal mammals (Diogo and Molnar 2014; Harper 2019; Dye 1987).

As a matter of fact, the following percentages, in order, turn out to be meaningful: 14.591% (end of the shortest sub-phase of Phase 1); 38.198% (end of Phase 1); 47.215% (minimum for the horizontal profile of the left-hand within Phase 2); 52.789% (end of the shortest sub-phase of the complement of 2S in Phase 2); 61.804% (end of Phase 2); 67.378% (maximum for the vertical profile of the dominant hand within Phase 3).

As already done for the knee flexion angle in the walking gait, taking the horizontal position of the non-dominant-hand (see also the technical remark 2 in Appendix B), the key idea is then to: 1. associate sewing points with the percentages 38.198%, 52.789%, 67.378%; 2. associate maximum, minimum, maximum points with the percentages 14.591%, 47.215%, 61.804% respectively; 3. use the five elementary time functions $a_{i}t^{2}+b_{i}t+c_{i}$, i=-1,0,1,2, for which jerk is minimized to zero (constant-acceleration sub-phase), to connect all the (consecutive) three sewing points and allow for jerk impulsive actions at the sub-phase transition instants; 4. carry out a ${\mathcal {C}}^{1}$-sewing of the elementary time functions to obtain an overall continuous function, with continuous derivative, that reconstructs the horizontal profile of the non-dominant-hand.

Also in this case, the resulting function, owing to the above alternation of maxima/minimum points and to the nature of the elementary basis functions, does have a shape that is similar to a biased sinus function multiplied by an exponential. It will be referred to as *golden faux-sinus* for tennis forehand and, by definition, is described by the system of equations (16)-(24) in Appendix A. Note that when tennis players artificially stop the non-dominant hand horizontal movement, the last two constraints are to be replaced, in analogy to walking, by the two constraints: A1) and A2) of Appendix A. Equations (16)-(22) and A1)-A2) thus constitute the block-hand-variant of (16)-(24) in Appendix A.

## Data analysis results

**Fig. 9:**
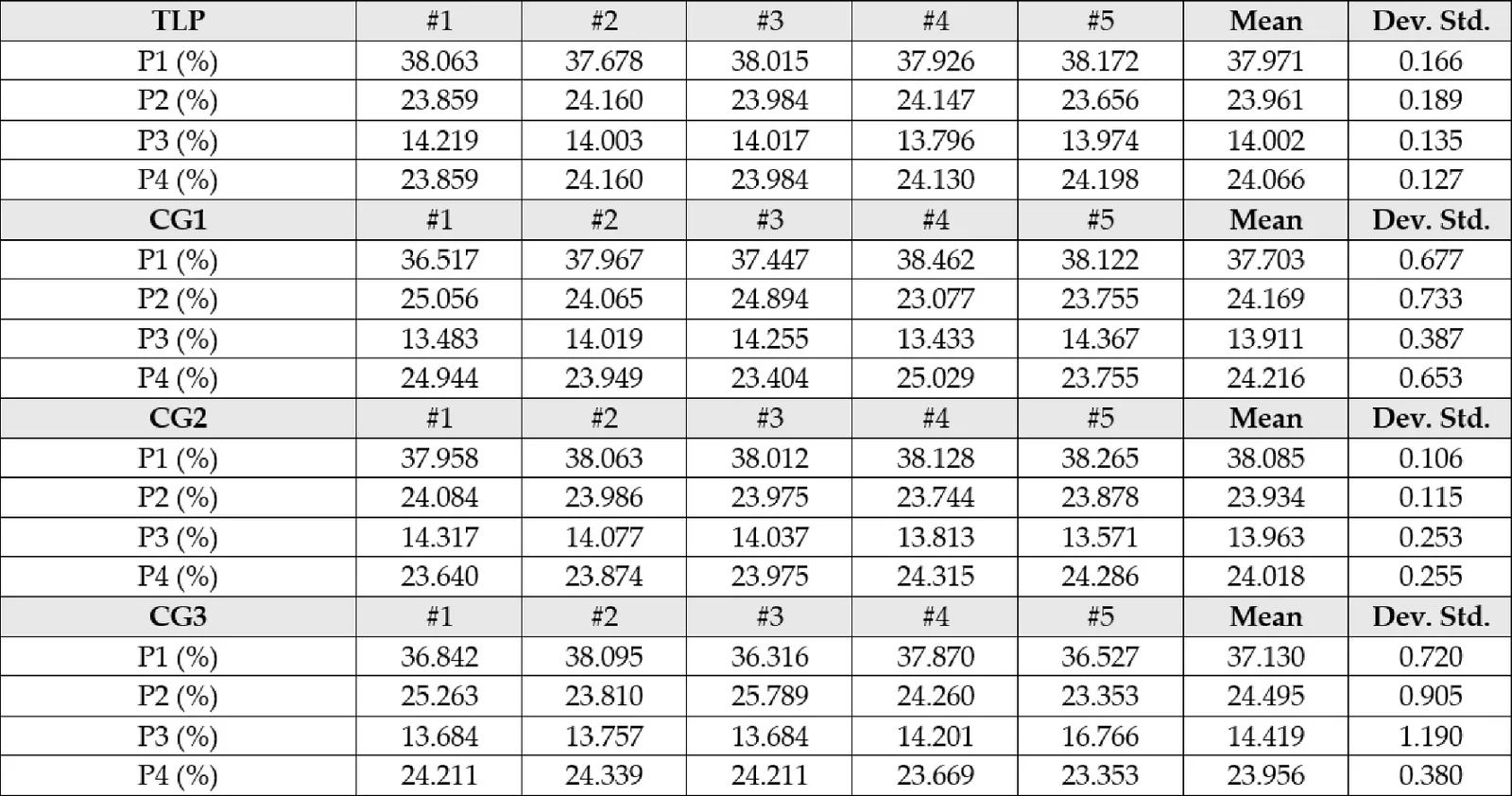
Subphase percentage durations for the top-level tennis players TPL, CG1, CG2, CG3.

**Fig. 10:**
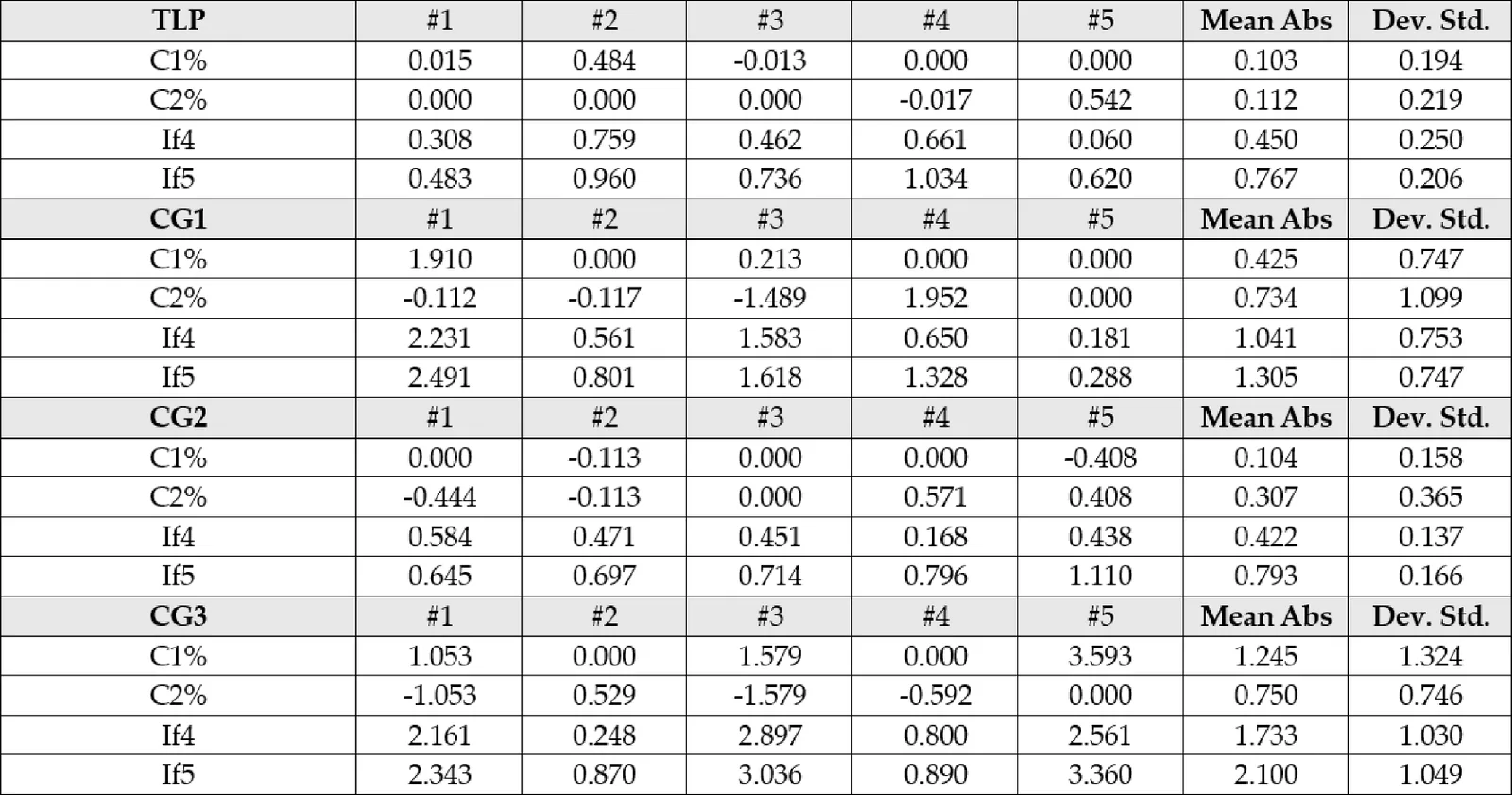
Percentage constraints and harmonicity indices (as defined in Section 3.3) for the top-level tennis players TPL, CG1, CG2, CG3.

Data in (Verrelli et al. 2025) from top-rank tennis players have already shown that a coordinatively mechanized and self-similarized stroke exists in real practice and, actually, in the challenging scenario of a training rally. On the other hand, the levels of coordinative mechanization and self-similarization were shown to increase (namely, the related indices decrease) with the quality of players: a tennis player first improves, over the stroke repetitions, the level of coordinative mechanization, and then increases the level of self-similarity. Here, the reader is instead referred to Figure 2 and Figures 9-10, in which (unreleased and totally original) time instants and sub-phase durations/self-similarity indices concerning five forehand strokes of the aforementioned top-level tennis players TLP, CG1, CG2, CG3 are reported. As a matter of fact, the quality of the forehand strokes of such top-level tennis players turns out to be so high that they are actually self-similar in time, even in a practice rally situation [CG3 also faces a scenario in which disturbing-like actions are present]. In particular, the entire sets of strokes are meaningfully very close to the self-similar partitioning nullifying indices ${\mathcal {I}}_{f,4}$ and ${\mathcal {I}}_{f,5}$. The conclusions of (Verrelli et al. 2025) are totally confirmed on the new top-level tennis players of this paper. Another point of strength is to recognize that the impact actually occurs each time very close to the predicted 0.61804% [TLP: 61.922%, 61.928%, 61.999%, 62.073%, 61.828%; CG1: 61.573%, 62.032%, 62.341%, 61.539%, 61.877%; CG2: 62.042%, 62.049%, 61.937%, 61.872%, 62.143%; CG3: 62.105%, 61.905%, 62.105%, 62.130%, 59.88%].

**Fig. 11:**
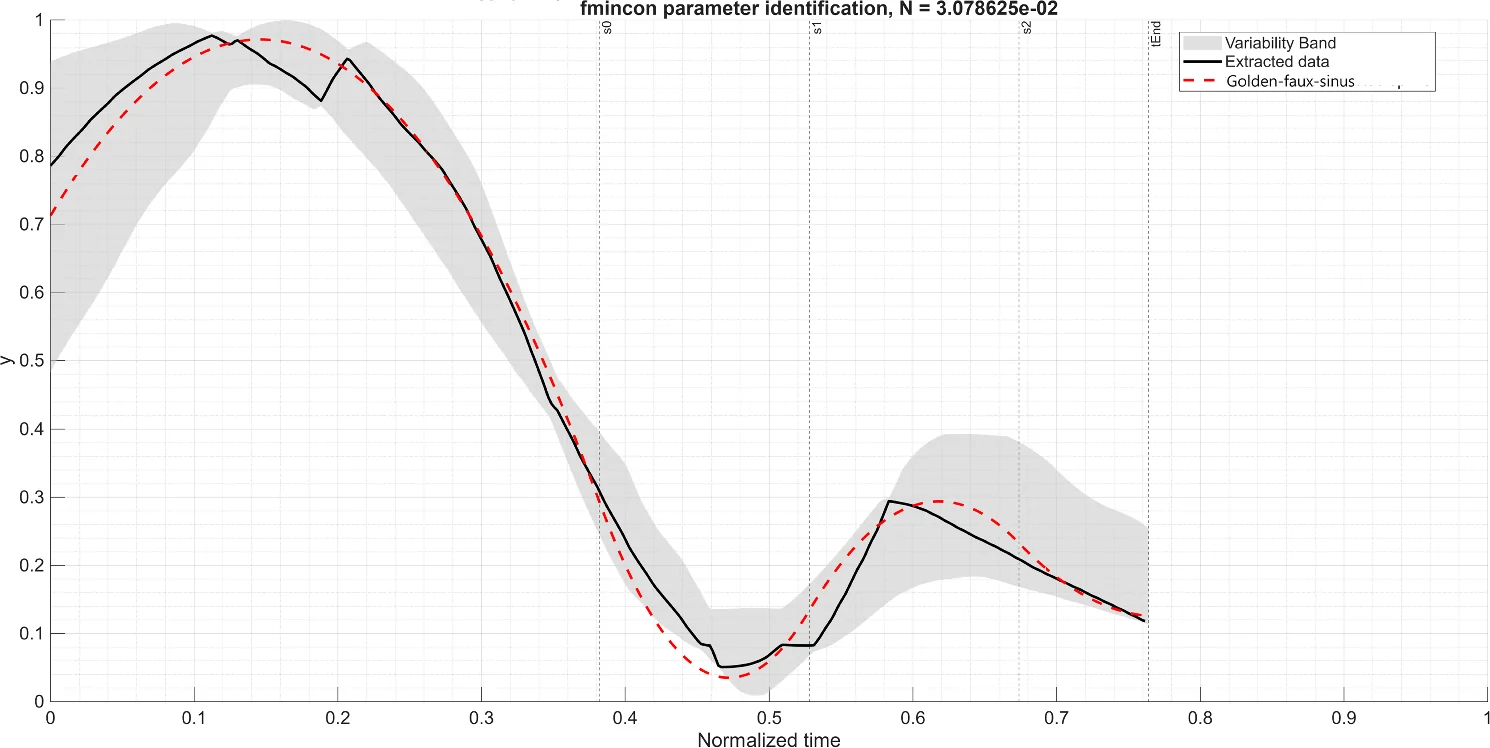
Normalized horizontal positions of the non-dominant hand (from 0 to 1) for the five forehand strokes of TLP (black curve representing the median) and *golden-faux-sinus* for tennis forehand (reconstructed profile by (16)-(24) of Appendix A) for their median [abscissa reporting the normalized time with respect to the total duration of the forehand stroke]. The gray band represents the interquartile range (IQR) variability

**Fig. 12:**
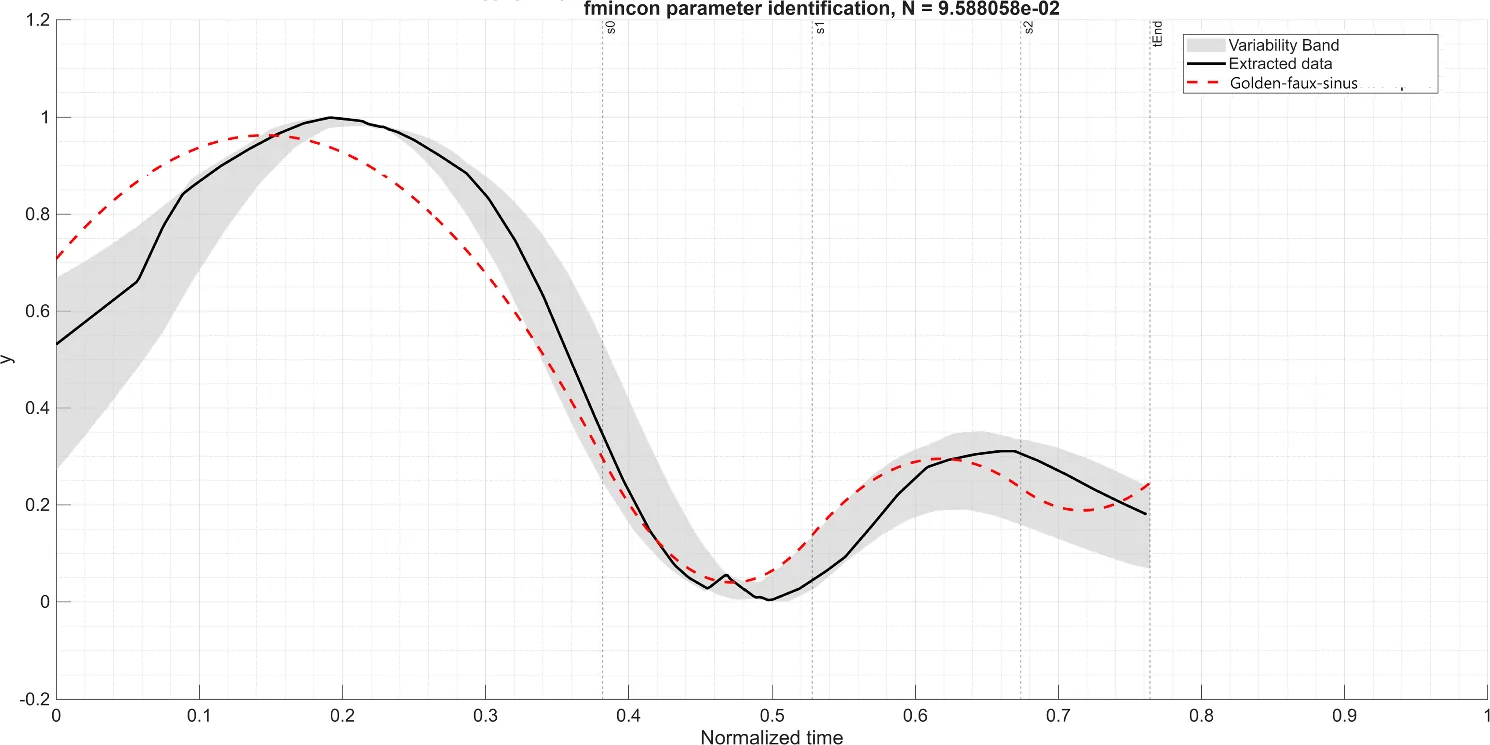
Normalized horizontal positions of the non-dominant hand (from 0 to 1) for the five forehand strokes of CG1 (black curve representing the median) and *golden-faux-sinus* for tennis forehand (reconstructed profile by (16)-(24) of Appendix A) for their median [abscissa reporting the normalized time with respect to the total duration of the forehand stroke]. The gray band represents the interquartile range (IQR) variability

**Fig. 13:**
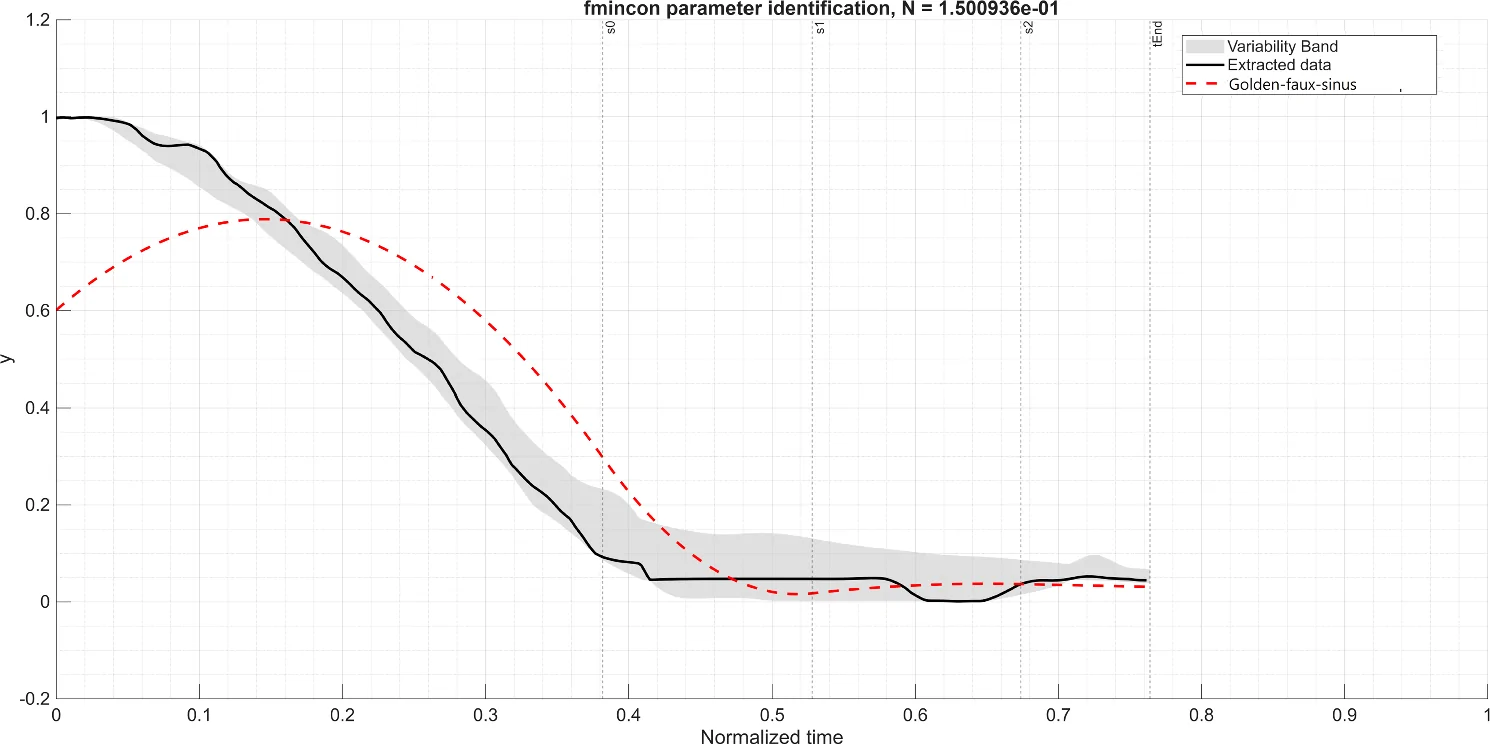
Normalized horizontal positions of the non-dominant hand (from 0 to 1) for the five forehand strokes of CG2 (black curve representing the median) and *golden-faux-sinus* for tennis forehand (reconstructed profile by (16)-(22) and A1)-A2) of Appendix A) for their median [abscissa reporting the normalized time with respect to the total duration of the forehand stroke]. The gray band represents the interquartile range (IQR) variability

**Fig. 14:**
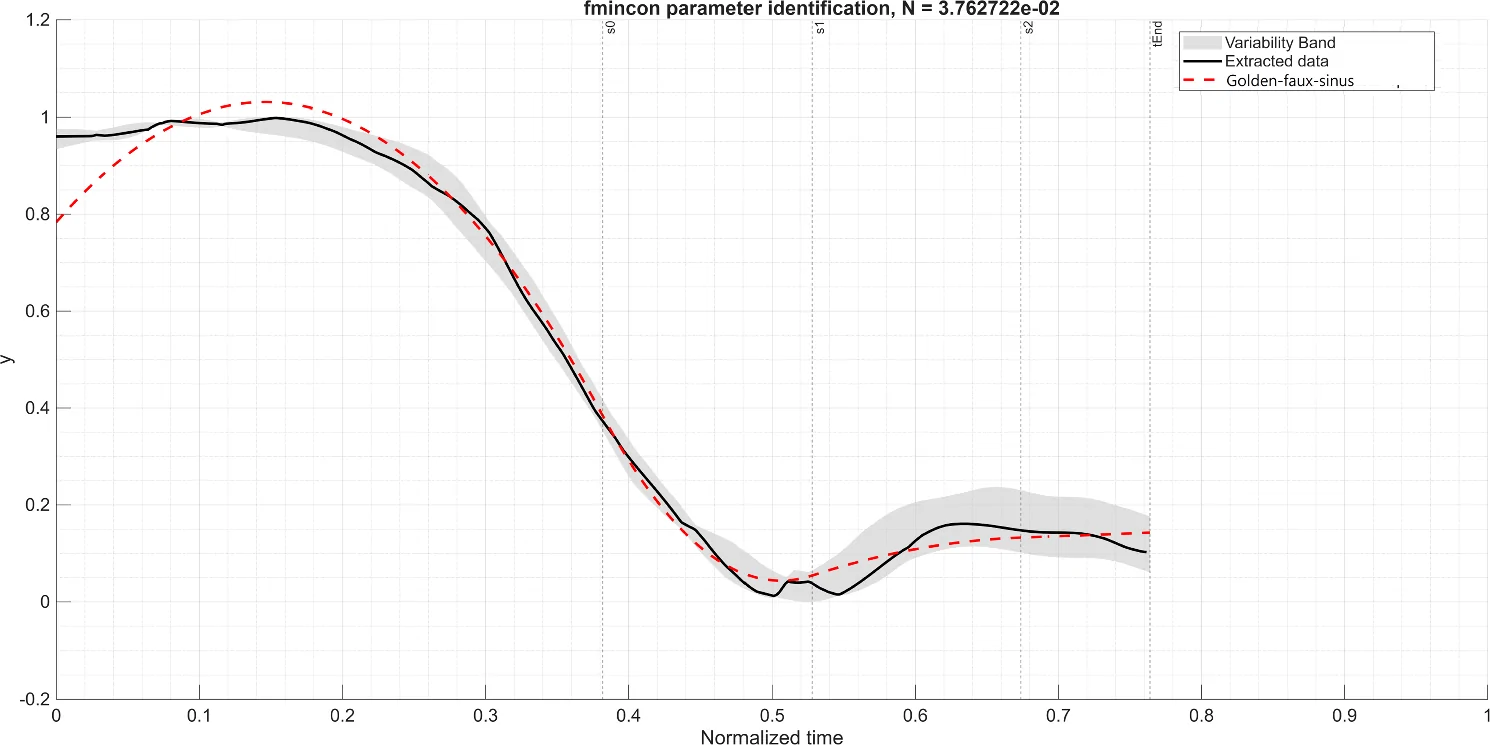
Normalized horizontal positions of the non-dominant hand (from 0 to 1) for the five forehand strokes of CG3 (black curve representing the median) and *golden-faux-sinus* for tennis forehand (reconstructed profile by (16)-(22) and A1)-A2) of Appendix A) for their median [abscissa reporting the normalized time with respect to the total duration of the forehand stroke]. The gray band represents the interquartile range (IQR) variability

**Fig. 15:**
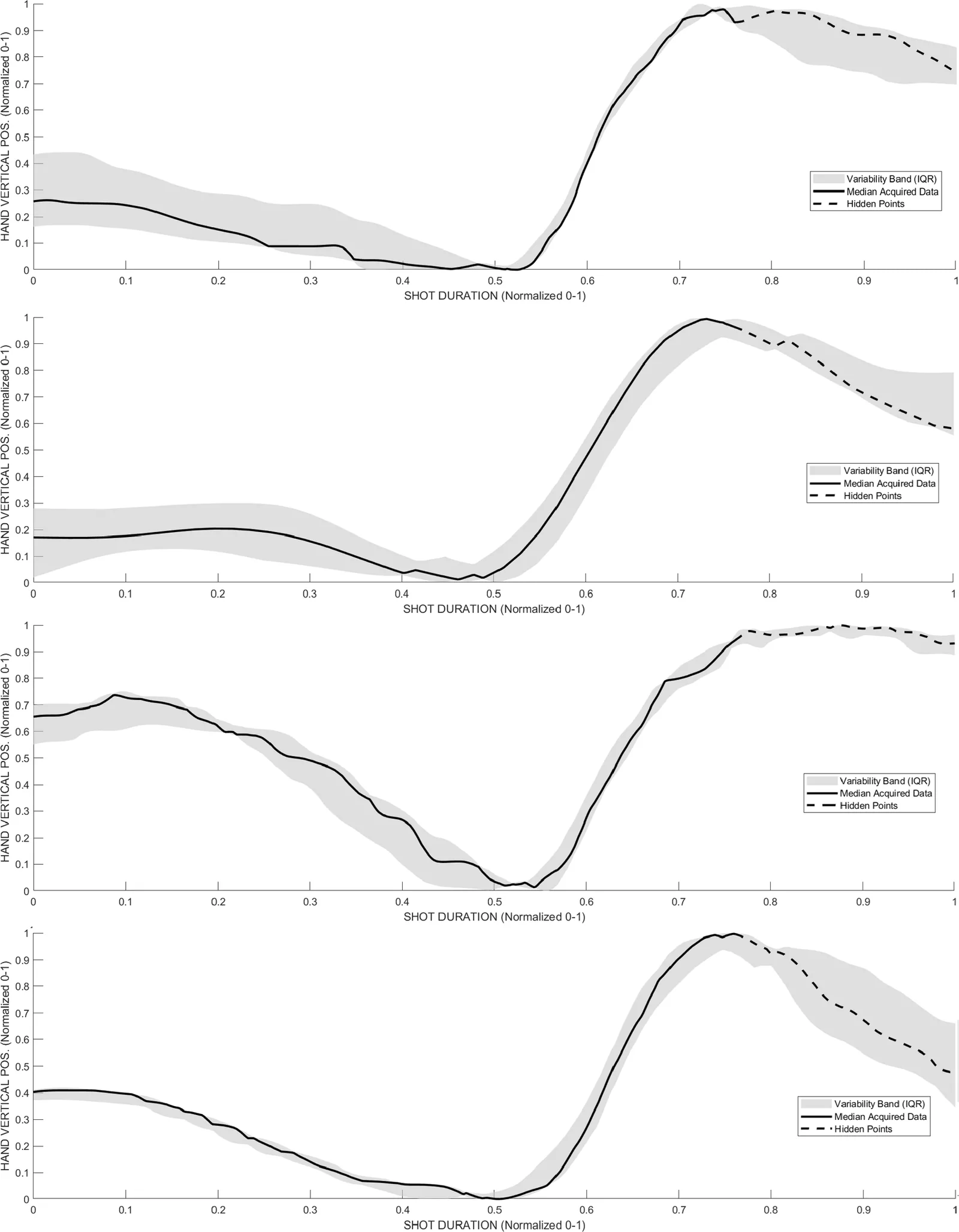
Normalized vertical positions of the dominant hand (from 0 to 1) for the five forehand strokes of TLP, CG1, CG2, CG3 (black curve representing the median) [abscissa reporting the normalized time with respect to the total duration of the forehand stroke. The gray band represents the interquartile range (IQR) variability

The reader is then referred to Figures 11-14, which represent, with reference to the partition of the stroke phase, the normalized horizontal positions of the non-dominant hand with reference to the strokes of Figure 2 and Figures 8-9 (the normalized vertical positions of the dominant hand are reported in Figure 15). It is worth emphasizing that the non-dominant hand (namely, the left hand for the subjects analyzed in this study) is the one that moves during the forehand stroke together with the rotational motion of the trunk (around the vertical axis). The trunk torsion, which is useful for the proper transfer of kinetic energy from the lower limbs to the upper limbs during the stroke (Roetert et al. 2009), dictates the temporal phases described in this study and previous ones (Landlinger et al. 2010). Indeed, in order to obtain useful information for the study of the forehand stroke using 2D video analysis, the movement of the non-dominant hand was decomposed into its two components within the image plane, and the main horizontal component was therefore examined while neglecting the vertical one. The reader can appreciate in those Figures 11-14 how the *golden faux-sinus* for tennis forehand coming from (16)-(24) of Appendix A or its variant (see also Appendix E) is able to reconstruct the medians of the non-dominant hand- normalized horizontal positions for the forehand stroke. The reconstruction errors are: N=0.030786, N=0.09528, N=0.15009, N=0.03762, respectively. Notice that the median is considered in place of the mean, since it is more robust and less affected by outliers.

The illustration provided by TLP and CG1, CG2, CG3 confirms that the optimization of the spatial profiles appears to be related to the moment of the player’s career, along with the inter-individual variations (Deghaies et al. 2025). From an ecological dynamics perspective (Araujo et al. 2019), here movement variability is not constituted by thes noise but by a functional adaptation to changing tasks and environmental constraints. Our findings are compatible with this view: the top-level player’s strokes showed moderate variability (CV 1–7%) while still approximating the golden ratio attractor. Thus, the *golden faux-sinus* should be interpreted as a mathematical attractor toward which the motor system gravitates, not as a fixed template that every stroke must exactly replicate.

**Fig. 16:**
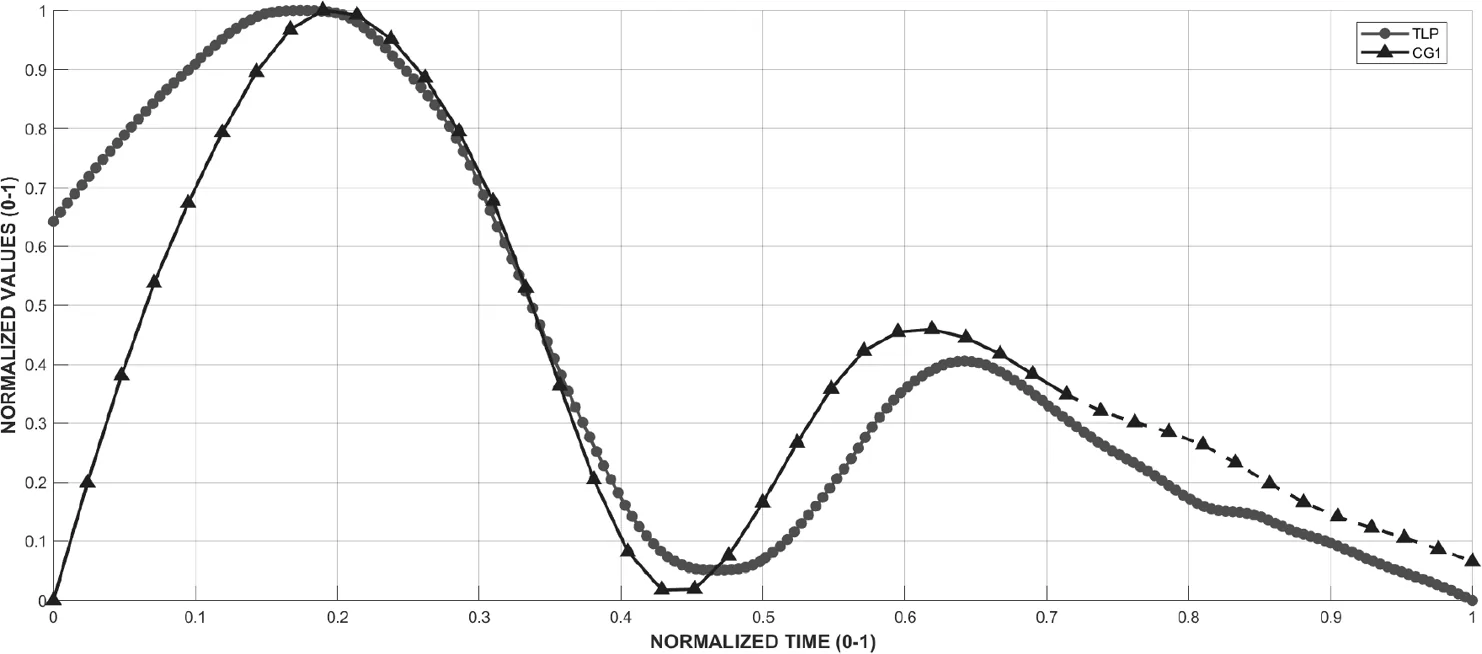
Normalized horizontal positions of the non-dominant hand (from 0 to 1) for the forehand stroke number 1 of TLP and number 5 of CG1 [abscissa reporting the normalized time with respect to the total duration of the forehand stroke].

Additionally, Figure 16 shows the normalized value of the horizontal displacement of the non-dominant hand during two forehand strokes by the same player taken one year apart. The two strokes with the most similar indices and coefficients and the highest level of self-similarity were selected in Figure 9 (TLP: stroke number 1; CG1: stroke number 5, 38.063-38.122, 23.859-23.755, 14.219-14.367, 23.859-23.755). Although the shots do not show marked differences in terms of timing, as can be seen in Figure 16 the normalized horizontal position of the non-dominant hand in CG1 (compared to TLP) introduces a tiny phase lead desynchronization between the normalized time for the second local maximum in the space profile and the normalized time for the impact (with greater effects also on the normalized time for the minimum in the space profile), as well as a phase lag desynchronization between the normalized time for the first local maximum in the space profile and the normalized time 0.14591. As already happened for healthy subjects walking at a comfortable speed, the second optimization principle acts differently from the first one.

## Discussion, forecast and limitations

Understanding how humans determine the timing of complex repetitive movements that are characterized by a high number of degrees of freedom is a challenging problem, since motor behavior is influenced by cognitive processes such as attention, perception, memory, and decision-making. Movement automaticity occurs when a subject no longer needs to pay attention to the act itself during the execution, with the natural and non-conscious consequent high-level coordination of a set of the body segments forming a smooth and efficient execution of the movement with its given sub-phases^4^. The performance is thus continuously shaped and refined until a stable pattern is formed, and the movement is naturally attracted to cover it. The original paper contribution has been to show that the *golden faux-sinus*, defined in accordance with the following:

### Definition

Given an ordered set Σ of specific T-SEM-induced instants characterizing the T-SEM-induced sub-phases of a coordinatively mechanized and self-similar (repetitive) human movement/gesture (including the walking gait or the forehand stroke) and given a space-time scalar funtion representing the movement of a limb or a part of it (along its dominant direction if multiple directions are involved), the *golden faux-sinus* is the function that comes from ${\mathcal {C}}^{1}$-connecting the images of the elements of a proper subset $\Sigma _s$ of Σ, through portions of (zero-jerk) second-degree polynomials of time *t* whose maxima/minima are attained at some elements of $\Sigma - \Sigma _{s}$,

accurately reconstructs a space-time profile of a limb or a part of it involved in the aforementioned movement/gesture. Such a definition introduces an enhanced version of the fluidity, in which the sewing points actually belong to an ordered set Σ of specific T-SEM-induced instants characterizing the T-SEM-induced sub-phases of a coordinatively mechanized and self-similar (repetitive) human movement/gesture.

In other words, the larger the number of T-SEM-induced sub-phases entering the self-similar sequence is, with the larger number of points of Σ entering the process, the larger the number of elementary functions connecting the sewing points is, and the more complex the movement results, though preserving the zero-jerk nature of the T-SEM-induced sub-phase and the jerk impulsive actions at the T-SEM-induced sub-phase transition instants. Now, movement smoothness, which jerk minimization corresponds to, is a pivotal parameter for assessing the quality of human motion, reflecting its fluidity and continuity (Caselli et al. 2025) (see also (Caprioli et al. 2024)). A movement is smooth when it is performed in a continuous fashion without any interruptions or peaks in its accelerations. Smoothness is often considered a positive characteristic feature of healthy and well-trained motor behaviour (Sejnowski 1998). Neural development in children (Berthier and Keen 2006), motor learning (Balasubramanian et al. 2012), and neurorehabilitation (Krebs et al. 1998) work to increase the smoothness of movements. The importance of performing smoothed movements is related to its intrinsic effect on effort minimization, sensorimotor control facilitation, and also multi-joint coordination (Harris and Wolpert 1998). This capacity of the sensorimotor control to synthesize and combine individual motor impulses into integral kinesthetic structures without any dystonia is also called kinetic melody Luria (1973). Despite its importance in sensorimotor control, smoothness of movement is often considered to be associated with the quality of movements, and some indices have been suggested for the assessment of smoothness of movements, such as the normalized jerk (Pascucci et al. 2024). Indeed, the results of this paper also move along the direction of the latest (Cocco et al. 2025), confirming that dual tasks have an overall deleterious effect on walking in patients with Parkinson’s Disease and highlighting the importance of the link between cognitive functions and motor skills. In particular, it has been shown that harmonicity indices (derived from Fibonacci-based temporal partitions) are sensitive to cognitive load in Parkinson’s disease patients, suggesting that the alteration of harmonicity – a sign of a desynchronization of the underlying motor mechanisms, in conditions of cognitive load – may capture motor-cognitive interactions. Our results extend this observation to the spatial domain, but further studies are needed to directly test whether spatial harmonicity (fit to the *golden faux-sinus) *is similarly affected by periods of nervous fatigue or exhaustion.

**Fig. 17:**
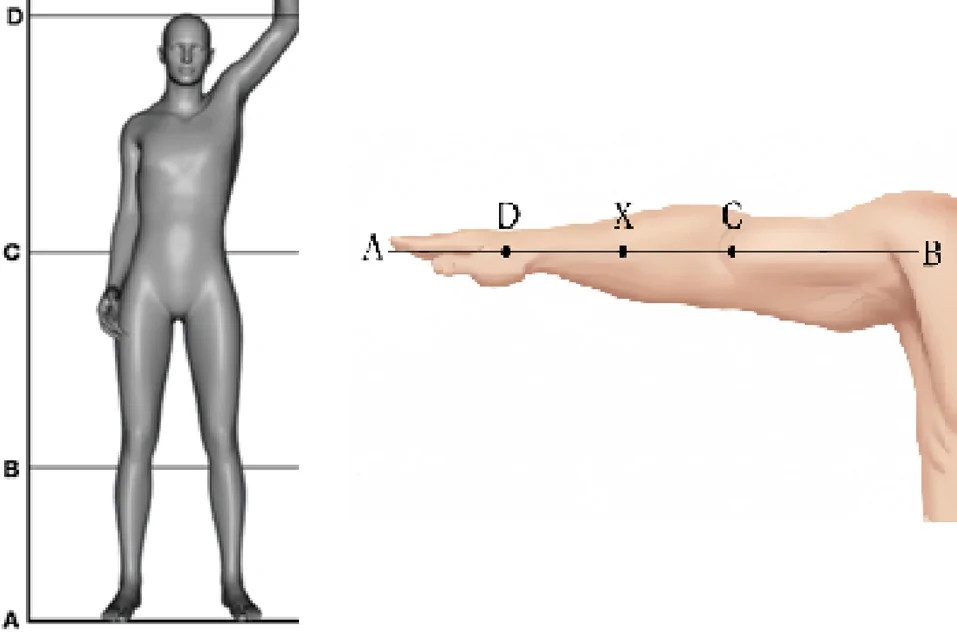
Human body and upper limb functional partition. Body: Head apex (D), L3 level (C), head of fibula level (B), ground level (A). Arm: point *X* as the middle point of the forearm transverse diameter, where it is becoming smaller.

Interestingly, the movement sub-phases, which the present study starts from and lead to two-dimensional harmonic patterns, are related to the actions of the main muscle groups involved in the execution of the stroke (Roetert and Kovacs 2019). This might be associated with the presence of the golden ratio in the involved body structures, from a functional point of view. It is worth mentioning (Iosa et al. 2016) and (Wang et al. 2017) (see Figure 17) and recognizing that the functional partition there discovered follows the more general rules reported in Appendix D. As far as walking is concerned, head apex (D), L3 level (C), head of fibula level (B), ground level (A) define segments composing the following 4th-length generalized Fibonacci sequence:

$$\begin{aligned} \textrm{Fib}_{4}: \overline{AB}, \overline{BC}, \overline{AC}, \overline{AD} \end{aligned}$$

under the extendability constraint $\overline{BC} = \overline{CD}$ with the last ratio $\overline{AD}/\overline{AC}=\phi$ imposing the chain of equalities $\overline{AC}/\overline{BC}=\phi$ and $\overline{BC}/\overline{AB}=\phi$. As far as the tennis forehand stroke (and front crawl swimming) is concerned, the functional partition of the upper limb (the point *X* is the middle point of the forearm transverse diameter, where it is becoming smaller) defines segments composing the following 5-th length generalized Fibonacci sequence:

$$\begin{aligned} \textrm{Fib}_{5}: \overline{DX}, \overline{XC}, \overline{AX}, \overline{XB}, \overline{AB} \end{aligned}$$

under the extendability constraints $\overline{AX} = \overline{CB}$, $\overline{XC} = \overline{AD}$ with the last ratio $\overline{AB}/\overline{XB}=\phi$ imposing the chain of equalities $\overline{XB}/\overline{AX}=\phi$, $\overline{AX}/\overline{XC}=\phi$, and $\overline{XC}/\overline{DX}=\phi$.

It is clear that, while the golden ratio appears in the anthropometric proportions of limb segments, joint rotations and segment dynamics are primarily governed by inertias and torques. Nevertheless, the functional partition shown in Figure 17 may reflect an underlying structural constraint that facilitates the generation of harmonic movements, a hypothesis that, however, requires direct dynamical testing.

From a scientific point of view, the *golden faux-sinus* model proposed in this paper offers a possible interpretative key for understanding cyclical movements, suggesting that the motor system spontaneously tends to converge towards solutions that combine temporal simplicity and spatial efficiency. By collecting the two domains into a composite one by the presence of the golden ratio in the involved body structures, we might interpret the optimization issues over the two domains in terms of resonant energy transfer from the kinetic chain to the ball, occurring with minimal muscular effort and maximum ballistic output (effortless power)^5^. All these observations may contribute to the development of new models capable of describing phenomena observed in different domains, such as locomotion, sports gestures, or complex rhythmic movements present in every expression of human movement. In practical terms, the idea of a double constraint –temporal and spatial – can be translated into quantitative tools useful for evaluating movement. The use of open-source software and of conventional video analysis demonstrates that high-precision kinematic evaluations can be performed without the need for extremely expensive motion capture laboratories, thus facilitating technology transfer to the field of athletic training. Indices based on a combination of temporal entropy and smoothness could offer a more complete measurement of movement quality, identifying deviations from the ideal structure at an early stage. This also has direct implications in the clinical field: pathological or non-optimized movements often show a deterioration in temporal regularity or an increase in spatial jerk, making these parameters potential sensitive markers for neuromotor disorders. On the other hand, markerless video applications (Edriss et al. 2025; Sampaio et al. 2024), wearable or acoustic sensors (Caprioli et al. 2025a, b), can be used in the assessment and training of these technical components. This way, the technical automation process could be made faster, more stable, and more resilient to the operating context.

Although this study is intended as a preliminary exploratory analysis to establish an original bridge between sports biomechanics and mathematical aesthetics and to provide an objective metric to evaluate movement quality, it has several limitations that deserve attention and have to be certainly addressed in future studies. First of all, the present approach relies on an emotional dimension of cybernetic aesthetics, claiming that movement patterns generate aesthetic feelings in the viewer. However, despite a notable absence of empirical data to validate this general claim in terms of perception scales or independent observer judgments (Rolf and Winkielman 2004), the manuscript assumes that beauty is an intrinsic property of the mathematical order of movement by relying on the golden ratio as a potential link between beauty and mathematical harmony of movements. On one hand, the golden ratio has been investigated by neuroscientists as an aesthetic pattern common in many different artworks across centuries, from the façade of the Parthenon to the Venus of Milo (Iosa et al. 2018), from Michelangelo’s Creation of Adam (Iosa et al. 2018) to the paintings of Rothko (Lucia et al. 2024). On the other hand, it was found in human anthropometry and human movements (Iosa et al. 2018). A human body with proportions following the golden ratio has been perceived as harmonic and beautiful by external observers (Nikgoo et al. 2009). Some results also support the idea that observers perceive as more aesthetically valued the walking of people when the ratio between the gait phase durations comply with the golden ratio (Bartolo et al. 2019).

A further consideration concerns the sample size. The detailed analysis was conducted on a small number of subjects and top-level tennis players. There is still a need to extend the verification of the model. In addition, the recordings of forehand strokes were obtained in ecological contexts and not in a controlled experimental environment. This is an advantage in terms of realism and robustness, but it introduces variables related to the playing scenario, the equipment, and the athlete’s condition that cannot be completely normalized. A more structured data collection could allow for more accurate isolation of the factors that influence the shape of the trajectory. A further limitation concerns the fact that the kinematic analysis of the sporting movement was conducted in two dimensions. Although the lateral plane captures much of the dynamics of the forehand, extending to three-dimensional studies would allow aspects such as joint rotations and out-of-plane movements to be explored, offering a more complete understanding of the principle of spatial optimization. In particular, the 3D analysis (e.g., joint rotations, racket orientation) shall be necessary for a complete validation. Finally, it should be noted that the *golden faux-sinus* represents an ideal mathematical model based on zero jerk and perfectly regular sewing conditions, with the number of key-elements of Σ playing a crucial role. In practice, actual movements may show a smaller number of key-elements of Σ being involved as well as slight deviations due to biomechanical constraints, inter-individual variations (Deghaies et al. 2025), or alternative motor strategies and players’ condition. The generalization of the model to less automated or more variable gestures will therefore require further study. It is, however, interesting to notice how similar shapes are traced by the vertical trajectory of the center of mass of tennis players during the serve (Deghaies et al. 2025).

## Conclusions

This paper has addressed the open problem of mathematically defining an attractor that unveils hidden coordinatively mechanized and self-similar aesthetical patterns appearing in cyclic human movements. The original contribution of this paper thus consists of illustrating how a second optimization dimension might be involved. Such a dimension is related to the movement space-shaping and fluidity enhancement through maxima-minima generation in the space domain with sub-phase jerk minimization & smooth transition among sub-phases, and leading to the *golden faux-sinus*. Such a function has been shown to be involved *via* experiments concerning top-level tennis players during some of the latest strongest moments of their career. Indeed, it integrates two complementary optimization principles: temporal entropy minimization (through Fibonacci-generated phase durations) and spatial smoothness (through zero-jerk sub-phases and jerk-impulsive transitions). From a cybernetic aesthetics perspective, this dual optimization reduces the informational redundancy of the movement, making its structure more predictable and thus more cognitively fluent for an observer. While direct spectator ratings were not collected, the model generates testable predictions for future experimental aesthetics studies concerning movements with lower temporal entropy and higher spatial smoothness. Future studies should analyze through surface electromyography sensors (EMGs) the muscle activation during the various sub-phases, to further deepen the analysis and allow for a broader understanding of the neuromuscular action underlying the harmonic gesture.

## Data Availability

Data supporting the findings of this study are available from the corresponding author upon reasonable request.

## Mathematical modeling (*golden faux-sinus*)

In accordance with Section 3.1, the *golden faux-sinus* for walking is defined by:

1 $$\begin{aligned}&a_{-1}t^{2}+b_{-1}t+c_{-1 | t=7.2955\%} = a_{0}t^{2}+b_{0}t+c_{0 | t=7.2955\%} \end{aligned}$$

2 $$\begin{aligned}&a_{0}t^{2}+b_{0}t+c_{0 | t=26.395\%} = a_{1}t^{2}+b_{1}t+c_{1 | t=26.395\%} \end{aligned}$$

3 $$\begin{aligned}&a_{1}t^{2}+b_{1}t+c_{1 | t=57.2955\%} = a_{E}t^{2}+b_{E}t+c_{E | t=57.2955\%} \end{aligned}$$

4 $$\begin{aligned}&a_{E}t^{2}+b_{E}t+c_{E | t=61.804\%} = a_{2}t^{2}+b_{2}t+c_{2 | t=61.804\%} \end{aligned}$$

5 $$\begin{aligned}&a_{2}t^{2}+b_{2}t+c_{2 | t=90.982\%} = a_{3}t^{2}+b_{3}t+c_{3 | t=90.982\%} \end{aligned}$$

6 $$\begin{aligned}&2a_{-1}t+b_{-1 | t=7.2955\%} = 2a_{0}t+b_{0 | t=7.2955\%} \end{aligned}$$

7 $$\begin{aligned}&2a_{0}t+b_{0 | t=26.395\%} = 2a_{1}t+b_{1 | t=26.395\%} \end{aligned}$$

8 $$\begin{aligned}&2a_{1}t+b_{1 | t=57.2955\%} = 2a_{E}t+b_{E | t=57.2955\%} \end{aligned}$$

9 $$\begin{aligned}&2a_{E}t+b_{E | t=61.804\%} = 2a_{2}t+b_{2 | t=61.804\%} \end{aligned}$$

10 $$\begin{aligned}&2a_{2}t+b_{2 | t=90.982\%} = 2a_{3}t+b_{3 | t=90.982\%} \end{aligned}$$

11 $$\begin{aligned}&2a_{0}t+b_{0 | t=11.804\%}= 0 \end{aligned}$$

12 $$\begin{aligned}&2a_{1}t+b_{1 | t=40.982\%}= 0 \end{aligned}$$

13 $$\begin{aligned}&2a_{2}t+b_{2 | t=76.395\%}= 0 \end{aligned}$$

14 $$\begin{aligned}&a_{-1}t^{2}+b_{-1}t+c_{-1 | t=0} = a_{3}t^{2}+b_{3}t +c_{3 | t=100\%} \end{aligned}$$

15 $$\begin{aligned}&a_{E}t^{2}+b_{E}t +c_{E | t=\bar{t}} = d \end{aligned}$$

where *d* is the value of the portion $a_{E}t^{2}+b_{E}t +c_{E}$ at any point $\bar{t} \in (57.2955\%,61.804\%)$ owing to the elastic return (*E* stands for elastic here). The above system of fifteen equations in fifteen unknowns (once $a_{-1}$, $b_{-1}$, $c_{-1}$, *d* are considered as known boundary conditions) admits a unique solution (the corresponding matrix is invertible and owning the determinant $8(p_1 - t_0)(p_2 - t_1)(p_3 - t_2)(p_4-t_E)(t_3 - 1)^2$, where $t_0 =0.072955$; $t_1 =0.26395$; $t_E=0.572955$, $t_2 =0.61804$; $t_3 =0.90982$; $p_1 =0.11804$; $p_2 =0.40982$; $p_3=0.76395$, $p_4=0.6$).

On the other hand, in accordance with Section 3.3 the *golden faux-sinus* for tennis forehand is defined by:

16 $$\begin{aligned}&a_{-1}t^{2}+b_{-1}t+c_{-1 | t=38.198\%} = a_{0}t^{2}+b_{0}t+c_{0 | t=38.198\%} \end{aligned}$$

17 $$\begin{aligned}&a_{0}t^{2}+b_{0}t+c_{0 | t=52.789\%} = a_{1}t^{2}+b_{1}t+c_{1 | t=52.789\%} \end{aligned}$$

18 $$\begin{aligned}&a_{1}t^{2}+b_{1}t+c_{1 | t=67.378\%} = a_{2}t^{2}+b_{2}t+c_{2 | t=67.378\%} \end{aligned}$$

19 $$\begin{aligned}&2a_{-1}t+b_{-1 | t=38.198\%} = 2a_{0}t+b_{0 | t=38.198\%} \end{aligned}$$

20 $$\begin{aligned}&2a_{0}t+b_{0 | t=52.789\%} = 2a_{1}t+b_{1 | t=52.789\%} \end{aligned}$$

21 $$\begin{aligned}&2a_{1}t+b_{1 | t=67.378\%} = 2a_{2}t+b_{2 | t=67.378\%} \end{aligned}$$

22 $$\begin{aligned}&2a_{-1}t+b_{-1 | t=14.591\%} = 0 \end{aligned}$$

23 $$\begin{aligned}&2a_{0}t+b_{0 | t=47.215\%}= 0 \end{aligned}$$

24 $$\begin{aligned}&2a_{1}t+b_{1 | t=61.804\%} = 0. \end{aligned}$$

The above system of nine equations in nine unknowns (once $a_{2}$, $b_{2}$, $c_{2}$ are considered as known boundary conditions) admits a unique solution (the corresponding matrix is invertible and owns the determinant $8(q_1 - s_1)(q_3 - s_0)(q_2 - s_2)$, where $s_0 =0.38198$;$s_1 =0.52789$; $s_2 =0.67378$; $q_1 =0.47215$; $q_2 =0.61804$; $q_3 =0.14591$). As declared in Section 3, when tennis players artificially stop the non-dominant hand horizontal movement, the last two constraints (23) and (24) are to be replaced, in analogy to (15) in walking that describes an elastic return constraint, by the following constraints: A1) $a_{0}t^{2}+b_{0}t+c_{0 | t=47.215\%}= d_{1}$ and A2) $a_{1}t^{2}+b_{1}t+c_{1 | t=61.804\%}= d_{2}$, with $d_{1}$ and $d_{2}$ being measured values. Equations (16)-(22) and A1)-A2) thus constitute the block-hand-variant of (16)-(24), with the corresponding matrix still being invertible and owning the determinant $2(q_1 - s_1)^{2}(q_3 - s_0)(q_2 - s_2)^{2}$.

## Technical Remarks

### Remark 1

The use of elementary time functions to shape & reconstruct the movements resembles – while extending – the approach in (Del Vecchio et al. 2003), which defines the *movemes* as primitives of motion. Here, the dynamical system generating each zero-jerk elementary function is $\dot{x}_{i} = [0,1,0;0,0,1;0,0,0,0]x_{i}$, $y_{i} = [1,0,0]x_{i}$ with initial condition $x_{i}(t_{i})=[c_{i},b_{i},2a_{i}]^\textrm{T}$.

### Remark 2

To confirm the assumption that different body dynamical compartments are connected in highly coordinated movements, if one portion of the knee flexion angle profile is neglected and the juxtaposition of the contro-lateral knee flexion angle profile from 0% to 50% of the gait cycle and of the knee flexion angle profile from 50% to 100% of the gait cycle is considered, it can be recognized that the resulting profile mimics the behaviour of a biased sinus function further multiplied by no exponential (namely, multipled by an exponential with almost zero rate). It does not yield the minimum and maximum points at 25% and 75%, but such minimum & maximum points are slightly delayed at 26.395% and 76.395%. The head profile in time of a swimming snake in (Gautreau et al. 2023), which is recognized to be a highly efficient movement, resembles such a function. Such a similarity is at the root of the key derivations of this paper. On the other hand, this is in accordance with the fact that, in the sagittal plane, the center of mass trajectory in walking is known to approximate a sinusoid, and the same happens in the frontal plane. In the sagittal plane, the movement is repeated at every stride, while in the frontal plane, at each couple of strides (right and left).

### Remark 3

Durations P3+P4, P1, P2, P3, P2S play the roles of the durations of the two swings, the double support, and the shortest sub-phase of the swing, the shortest sub-phase of the double support, respectively, for the walking gait.

### Remark 4

As for the walking gait, the juxtaposition of the horizontal profile of the left hand from 0% to its minimum value and of the vertical racquet-hand profile (on the camera plane) for the remaining part mimics the considerations reported in Remark 2. In this light, the hand profiles of Figures 11-14 can be viewed as the mirrored counterpart of the knee profile of Figures 3-5.

### Remark 5

As commonly happens for the walking gait and the tennis forehand, i) the knee flexion angle profile and the contro-lateral knee flexion profile, both considered from 0% to 50% of the gait cycle, exhibit a mirrored association between sewing points and extremum points, excepting for the normalized times 0.572955 and 0.072955 to which sewing points correspond for both the profiles; ii) the horizontal profile of the left hand from its minimum value to the 100% of the stroke and the vertical profile of the racquet-hand over the same domain, exhibit a mirrored association between sewing points and extremum points, excepting for the normalized times 0.38198 and 0.67378 to which sewing points correspond for both the profiles. It shows how the golden temporal partition meaningfully affects both the links in the movement execution.

### Remark 6

The stroke duration, left free from the temporal optimization, has a mirrored counterpart in the initial shape of the time-profile in the space, left free by the spatial optimization. Additional optimization principles working on such free quantities are not excluded.

### Remark 7

Notice how the statement of Proposition 1 can be aesthetically illustrated by means of a regular pentagon, by repetitively associating, in a straightforward fractal fashion, the elements of the generalized Fibonacci sequence therein with the lengths of the diagonals and sides of each of the three sub-pentagons (called internal innermost, innermost and inner pentagons hereafter) generated by the diagonals, sub-diagonals, and sub-sub-diagonals of a star pentagon, In other words, if one just takes a regular pentagon with unitary diagonal, then: P3S is the diagonal of the internal innermost pentagon; P2S is the side of the innermost pentagon and P3 is its diagonal; P2 is the side of the inner pentagon and P3+P4 is its diagonal; P2+P1 is the side of the pentagon and 1 is its diagonal.

## Video-analysis and inter-reliability

A sample of images is used to study the inter-rater reliability of the measurement system. The difference in measurements for the X and Y coordinates, the Euclidean marker position error, and the percentage error are computed for both the raw data and the interpolated signal. Nonparametric tests are used to increase the power of the statistics. The Wilcoxon Signed-Rank Test, Intraclass Correlation Coefficient (ICC) (Koo and Li 2016; Shrout and Fleiss 1979), and Spearman’s correlation ρ (Xu et al. 2013) are computed to determine the agreement of the measurements carried out by the two raters. Effect sizes (ES) given by the rank-biserial correlation are derived (a small ES is 0.10–0.29, moderate 0.30–0.49, and large >0.50). The statistical analysis is carried out by resorting to JASP (Version 0.18.3) and R software (Version 4.4.2). The signals obtained are normalized on a time scale from 0 to 100, and in amplitude from 0 to 1. The signals derived from 160 couples of images are compared to assess the inter-rater reliability (Tables 1, 2).

**Table 1:** Differences between Measure 1 (M1) and Measure 2 (M2) of X and Y coordinate in raw and interpolated signals.

**Raw signal (n = 160)**		**Interpolated signal (n = 199)**	
Difference X ( M1 - M2)	-1.54 ± 3.4 px	Difference X ( M1 - M2)	-0.27 ± 4.1 px
Difference Y ( M1 - M2)	-0.12 ± 2.2 px	Difference Y ( M1 - M2)	0.49 ± 2.4 px
Euclidean Position Error	3.4 ± 2.7 px	Euclidean Position Error	3.9 ± 2.8 px
Euclidean Percentage Error	2.0 ± 1.8 %	Euclidean Percentage Error	2.5 ± 1.9 %

**Table 2:** Inter-rater agreement between Measure 1 and Measure 2 of X and Y coordinates in raw and interpolated signals. IQR, inter-quartile range; ρ, Spearman’s correlation coefficient; ICC, Intraclass Correlation Coefficient; 95% Confidence Interval (CI) for ICC; Wilcoxon Signed-Rank (WSR) t-test; ES, Effect size by the Rank-Biserial Correlation; and 95% CI for ES.*** indicates a *p*-value < 0.001 in Spearman’s correlation.

**Raw signal (n = 160)**							
**Differences**	ρ	**ICC3,1**	**95% CI ICC**	***WSR***		**ES**	**95% CI ES**
				W	p		
X Measures	0.995***	0.996	0.995 to 0.997	3526	< 0.001	-0.452	-0.583 to -0.300
Y Measures	0.982***	0,995	0.994 to 0.997	7000	40	0.087	-0.091 to 0.260

**Interpolated signal (n = 199)**							
**Differences**	ρ	**ICC3,1**	**95% CI ICC**	***WSR***		**ES**	**95% CI ES**
				W	p		
X Measures	0.995***	0.838	0.791 to 0.875	9407	0.505	-0.055	-0.212 to 0.105
Y Measures	0.990***	0,996	0.994 to 0.997	13217	< 0.001	0.328	0.179 to 0.463

## Back to (Verrelli et al. 2025)

A Fibonacci sequence is one of the simplest ways of generating a sequence on the basis of a self-referential loop: each element is the sum of the two elements that precede it. Starting from 0 and 1, the sequence begins as 0, 1, 1, 2, 3, 5, 8, 13, 21, 34, whereas starting from 1 and 1, the sequence begins as 1, 1, 2, 3, 5, 8, 13, 21, 34, 55. Now, if the elements of the second sequence above are multiplied by a positive real α and the elements of the first sequence above by a positive real $\beta -\alpha$, then the sequence given by the sum of the two resulting sequences reads $\alpha , \beta , \alpha +\beta , \alpha +2\beta , 2\alpha +3\beta , 3\alpha +5\beta , 5\alpha +8\beta , 8\alpha +13\beta , 13\alpha +21\beta , 21\alpha +34\beta$, that is a generalized Fibonacci sequence with seeds α and β. Such a relaxation provides the analyst with larger degrees of freedom if the elements of the Fibonacci sequences have to refer to the durations of the subphases of a physical movement or gesture that naturally differ from 0 and 1. Now, consider an event ${\mathcal {F}}$ – here consisting, in a first approximation – of three consecutive disjoint phases ${\mathcal {F}A}$, ${\mathcal {F}B}$, ${\mathcal {F}C}$ with durations $\textrm{d}_{\mathcal {F}A}$, $\textrm{d}_{\mathcal {F}B}$, $\textrm{d}_{\mathcal {F}C}$. The duration $\textrm{d}_{\mathcal {F}}$ of the event ${\mathcal {F}}$ thus satisfies $\textrm{d}_{\mathcal {F}} = \textrm{d}_{\mathcal {F}A} + \textrm{d}_{\mathcal {F}B} + \textrm{d}_{\mathcal {F}C}$. Without loss of generality, let ${\mathcal {F}A}$ be the phase with the minimum duration. The generalized (non-decreasing) Fibonacci sequence of length 3 associated with the aforementioned partition is given by: $\textrm{Fib}_3: \textrm{d}_{\mathcal {F}C}, \textrm{d}_{\mathcal {F}A} + \textrm{d}_{\mathcal {F}B}, \textrm{d}_{\mathcal {F}}$. The main question is: under which conditions, the length of the above Fibonacci sequence $\textrm{Fib}_3$ can be increased by splitting, into the two phases ${\mathcal {F}A}, {\mathcal {F}B}$, the aggregate phase ${\mathcal {F}A} \cup {\mathcal {F}B}$ that appears, with its duration, as a second element of $\textrm{Fib}_3$? Answering this question led to the following:

### Definition 1

Event ${\mathcal {F}}$ above, consisting of three consecutive disjoint phases ${\mathcal {F}A}$, ${\mathcal {F}B}$, ${\mathcal {F}C}$ whose durations compose the Fibonacci sequence $\hbox {Fib}_{3}$, is *once-Fibonacci-left-extendable* if it is symmetrically partitioned, i.e., if the second and the first elements of (3) satisfy the temporal constraint $\textrm{d}_{\mathcal {F}B} = \textrm{d}_{\mathcal {F}C}$.

In other words, a symmetrically partitioned event ${\mathcal {F}}$ consists of a shorter phase ${\mathcal {F}A}$ and two longer phases ${\mathcal {F}B}$, ${\mathcal {F}C}$ of equal duration. The resulting pattern, which symmetry is at the root of, constituted the special pattern that the automatic generation process aims at replicating, while the length of the Fibonacci sequence is increased from 3 to 4 (namely, step 3-4), as the following theorem showed.

### Theorem 1

The generalized (non-decreasing) Fibonacci sequence of length 4 (extending $\hbox {Fib}_{3}$ to the left): $\textrm{Fib}_4: \textrm{d}_{\mathcal {F}A}, \textrm{d}_{\mathcal {F}C}, \textrm{d}_{\mathcal {F}A} + \textrm{d}_{\mathcal {F}C}, \textrm{d}_{\mathcal {F}}$ is associated with the once-Fibonacci-left-extendable event ${\mathcal {F}}$ of Definition 1.

The same logical scheme (Definition-Theorem) previously reported naturally extends to any step *r*-r+1. Coming back to the generalized Fibonacci sequence $\textrm{Fib}_{4}$: *a*, *b*, *c*, *d* of Theorem 1 (the same reasoning can be easily extended to Fibonacci sequences of larger length), with $\textrm{d}_{\mathcal {F}A}= a$, $\textrm{d}_{\mathcal {F}C}= b$ and $\textrm{d}_{\mathcal {F}A} + \textrm{d}_{\mathcal {F}C}= c$, $\textrm{d}_{\mathcal {F}}= d$, take the three differences $d_{1}=b/a-c/b$, $d_{2}=d/c-c/b$, $d_{3}=b/a-d/c$ and let $M_{R}$ be a sufficiently large positive odd integer. Let ${\mathcal {P}}$ be a finite partition of the compact set $[-M_{R},M_{R}]$, with disjoint blocks (or cells) ${\mathcal {A}}_{j}$ of the form:

$$\begin{aligned} {\mathcal {A}}_{j}= &  [x_{j},x_{j+1}), \ \ \ j=1,2, \ldots , M_{R}-1, \\ {\mathcal {A}}_{j}= &  [x_{j},x_{j+1}], \ \ \ j=M_{R}, \end{aligned}$$

where $x_{j+1}=x_{j}+2$, $j=1,2,\ldots , M_{R}$, and $x_{1}=-M_{R}$. Let ${\mathcal {P}}_{l}$ a finite refinement of ${\mathcal {P}}$ ($l=0,1,\dots ,R_{l}$, $R_{l}$ is a sufficiently large natural number), with finer blocks ${\mathcal {A}}_{k[j]}^{[l]}\subset {\mathcal {A}}_{j}$ of the form:

$$\begin{aligned} {\mathcal {A}}_{k[j]}^{[l]}= &  [x_{k[j]}^{[l]}, x_{k+1[j]}^{[l]}), \end{aligned}$$

where $x_{k+1[j]}^{[l]}=x_{k[j]}^{[l]}+1/2^{l}$, $k=1,\ldots ,2^{(l+1)}$, and $x_{1[j]}^{[l]}=x_{j}$. For each $l=0,1,\ldots ,R_{l}$, define the set of characters (or letters)

$$\begin{aligned} \Sigma ^{[l]}=\{x_{1}=x_{1[1]}^{[l]},\ldots ,x_{2^{(l+1)}+1[1]}^{[l]}=x_{2}=x_{1[2]}^{[l]},\ldots , \ldots \}. \end{aligned}$$

Consider the string of characters: $(s_{1}^{[l]},s_{2}^{[l]},s_{3}^{[l]})$, where $s_{1}^{[l]}$, $s_{2}^{[l]}$, $s_{3}^{[l]}$ belong to the above set $\Sigma ^{[l]}$ and $s_{m}^{[l]}$ (m=1,2,3) equals the smallest element of ${\mathcal {A}}_{k[j]}^{[l]}$ when the difference $d_{m}$ belongs to the block ${\mathcal {A}}_{k[j]}^{[l]}$. Let $p_{*r}^{[l]}$ be the number of characters belonging to the *r*-th character type in the three-elements-string $(s_{1}^{[l]},s_{2}^{[l]},s_{3}^{[l]})$ divided by 3 ($r=1,\ldots ,N^{[l]}$, $N^{[l]} \le 3$). Finally take the Shannon index for such a string $(s_{1}^{[l]},s_{2}^{[l]},s_{3}^{[l]})$ as

$$\begin{aligned} {\mathcal {H}}_{s}^{[l]}= &  - \sum _{r=1}^{N^{[l]}}p_{*r}^{[l]}\textrm{ln}\left( p_{*r}^{[l]}\right) , \end{aligned}$$

where $\sum _{r=1}^{N^{[l]}}p_{*r}^{[l]}=1$. The more unequal the abundances of types in the string is, the smaller the corresponding Shannon entropy is made, with ${\mathcal {H}}_{s}^{[l]}$ satisfying

$$\begin{aligned} {\mathcal {H}}_{s}^{[l]} \in \left[ 0, \textrm{ln}\left( N^{[l]}\right) \right] . \end{aligned}$$

In particular, if all abundance is concentrated to one type, Shannon entropy is zero and there is no uncertainty in predicting the type of the next entity. The case in which the golden ratio ϕ is a fixed point for the consecutive ratios *b*/*a*, *c*/*b* and *d*/*c* is thus characterized by the condition $\sum _{l=1}^{R_{l}}{\mathcal {H}}_{s}^{[l]}=0$, for any $R_{l} \in \mathbb {N} \cup \{ 0 \}$.

## Reconstruction procedure

The parameters of the *golden-faux-sinus* (including the set of the boundary conditions) have been identified by minimizing the normalized fitting error using the constrained nonlinear optimization (Matlab) function fmincon, allowing the reconstructing-curve to best approximate the experimental data. The objective function has been defined as the normalized fitting error: $N = \Vert y_\textrm{fit} - y_\textrm{data} \Vert / \sqrt{(}n)$, where $y_\textrm{data}$ is the extracted experimental curve, $y \textrm{fit}$ is the reconstructing-curve from the current parameter values, and *n* is the number of samples. The optimization thus aims to find the parameter values that minimize *N*. In particular, fmincon iteratively updates the unknown parameter vector starting from an initial guess and searching for the values that minimize the fitting error. Constraints – such as -$b_{-1}$/2$a_{-1}$ ≥ 0.11804 in the *golden-faux-sinus* for walking – can be introduced as well.

## Footnotes

1 Preliminary results on human walking have been presented at the 13th INTERNATIONAL NEW YORK CONFERENCE ON EVOLVING TRENDS IN INTERDISCIPLINARY RESEARCH & PRACTICES, March 15-18, 2026, Manhattan, New York City. Partial results on tennis forehand have been presented at the WORLD TENNIS CONFERENCE WTC6, hosted by the GPTCA/Segal Institute and supported by the ATP.

2 In particular, each healthy subject carries out, at a comfortable speed, a 20-meter walking test while wearing wearable sensors of the motion capture system Movit System G1 (Captiks, Rome, Italy) for movement capture and analysis (El Arayshi et al. 2022). The Movit System G1 provides accelerometer, gyroscope, magnetometer, quaternion, and barometer synced data, and is composed of 13 DOF wireless light-weight wearable small inertial devices and a USB wireless receiver. The Motion Studio & Motion Analyzer software, used in conjunction with the aforementioned data acquisition system, is then, respectively, used to collect the movement data from each sensor and fuse/process sensor data.

3 To this purpose, consider the front crawl partition in Verrelli et al. (2021b): entry-and-stretch phase ES, which starts from the hand entering the water until it reaches the most frontal position of its trajectory; downsweep-and-catch phase DC, in which the hand descends vertically after the stretch phase in the sagittal plane; insweep phase IN, in which the hand, at the end of the downsweep phase, moves internally from left to right in the frontal plane; upsweep phase UP, which can be identified as the ascent phase in the sagittal plane; exit phase EX, in which the hand comes out from the water; recovery phase RE, which starts when the arm exits the water and ends when the arm drops back into the water.

4 As aforementioned, the number of distinct sub-phases of the movement is naturally associated with the complexity of the movement itself and to its space-shape.

5 Pushing a person in a swing in time with the swing resonant frequency makes the swing go higher and higher, while attempts to push the swing at a faster or slower tempo produce smaller arcs. Perceiving a resonant pushing action is immediate, with respect to a non-resonant one.
